# General Principles Underpinning Amyloid Structure

**DOI:** 10.3389/fnins.2022.878869

**Published:** 2022-06-02

**Authors:** Alexander I. P. Taylor, Rosemary A. Staniforth

**Affiliations:** School of Biosciences, University of Sheffield, Sheffield, United Kingdom

**Keywords:** amyloid structure, cryo-EM, ssNMR, protein aggregation, protein folding, neurodegeneration, steric zipper, amide ladder

## Abstract

Amyloid fibrils are a pathologically and functionally relevant state of protein folding, which is generally accessible to polypeptide chains and differs fundamentally from the globular state in terms of molecular symmetry, long-range conformational order, and supramolecular scale. Although amyloid structures are challenging to study, recent developments in techniques such as cryo-EM, solid-state NMR, and AFM have led to an explosion of information about the molecular and supramolecular organization of these assemblies. With these rapid advances, it is now possible to assess the prevalence and significance of proposed general structural features in the context of a diverse body of high-resolution models, and develop a unified view of the principles that control amyloid formation and give rise to their unique properties. Here, we show that, despite system-specific differences, there is a remarkable degree of commonality in both the structural motifs that amyloids adopt and the underlying principles responsible for them. We argue that the inherent geometric differences between amyloids and globular proteins shift the balance of stabilizing forces, predisposing amyloids to distinct molecular interaction motifs with a particular tendency for massive, lattice-like networks of mutually supporting interactions. This general property unites previously characterized structural features such as steric and polar zippers, and contributes to the long-range molecular order that gives amyloids many of their unique properties. The shared features of amyloid structures support the existence of shared structure-activity principles that explain their self-assembly, function, and pathogenesis, and instill hope in efforts to develop broad-spectrum modifiers of amyloid function and pathology.

## 1. Introduction

**Figure 1:**
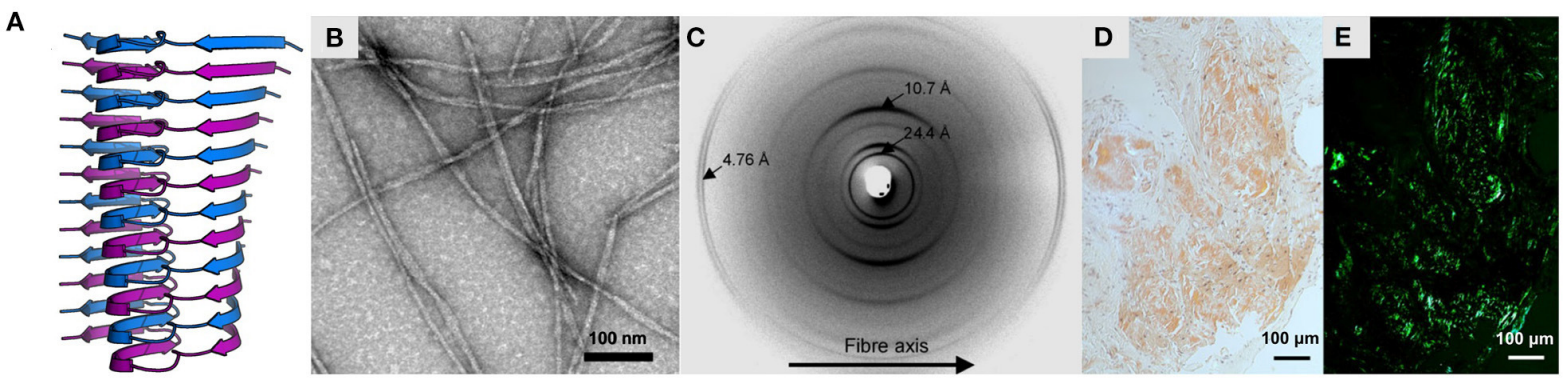
Characteristics of amyloid fibrils. **(A)** Structure of Aβ(1-42) fibrils produced *in vitro*, obtained by solid-state NMR spectroscopy (PDB ID: 2mxu; Xiao et al., 2015). The structure is shown as a ribbon diagram, with stacked monomeric subunits alternately colored blue and purple. **(B)** Negative-stain electron micrograph of the same fibrils, adapted with permission from Xiao et al. (2015). **(C)** X-ray fiber diffraction pattern of partially aligned amyloid fibrils formed by the KFFEAAAKKFFE peptide, reproduced with permission from Makin et al. (2005) (Copyright 2005 National Academy of Sciences). **(D,E)** Light microscopy images of light chain amyloid stained with Congo red dye, under **(D)** normal illumination and **(E)** polarized light, reproduced with permission from Swuec et al. (2019). Note the green birefringence under polarized light, indicative of cross-β structure.

Amyloids are fibrous assemblies of protein with a characteristic cross-β structure, consisting of a continuous, extensive, ribbon-like intermolecular β-sheet (Figures 1A,B). Amyloids have a distinctive set of structural and functional properties, including a high degree of molecular order, unusual stability and tensile strength, and the capacity to replicate their conformation indefinitely by self-templating and seeding. Diagnostic features of amyloids include an X-ray fiber diffraction pattern with an intense meridional reflection at ~4.7 Å (Astbury et al., 1935; Eanes and Glenner, 1968; Figure 1C), Congo red birefringence (Bennhold, 1922; Ladewig, 1945; Figures 1D,E), and thioflavin T (ThT) binding-induced fluorescence (LeVine, 1993). Due to their stability, capacity for uncontrolled self-replication, and ability to induce further protein misfolding, amyloids are often pathogenic, and their formation is associated with over fifty disorders, including Alzheimer's disease (Glenner and Wong, 1984), Parkinson's disease (Spillantini et al., 1997), and Huntington's disease (Perutz, 1999). At the same time, it has become clear that the capacity for amyloid formation is a universal or near-universal feature of polypeptide chains, and cross-β structure has been induced in many otherwise non-amyloidogenic proteins (Astbury et al., 1935; Guijarro et al., 1998; Litvinovich et al., 1998; Chiti et al., 1999; Fändrich et al., 2001), homopolypeptides (Fändrich and Dobson, 2002), and non-polypeptide amphiphilic polymers (Bradford and Iverson, 2008). Moreover, at physiological concentrations, amyloids are the most stable conformational state for many proteins, meaning that the native state is often a metastable phenomenon (Baldwin et al., 2011; Varela et al., 2018). Given their stability, universality, and capacity for self-directed assembly, it is unsurprising that biology has repeatedly harnessed amyloids to perform functional roles, such as in bacterial cell adhesion (Chapman et al., 2002), human melanin biosynthesis (McGlinchey et al., 2009), and, intriguingly, even memory (Shorter and Lindquist, 2005; Krüttner et al., 2012). Similarly, the long-range molecular order and favorable mechanical properties of amyloids make them highly attractive for the development of nanomaterials, such as scaffolds for catalysts, templates for nanoparticles, and novel adhesives (Nguyen et al., 2014; Zhong et al., 2014; Al-Garawi et al., 2017). Thus, structural studies of amyloids can provide crucial insights into amyloid-related pathology, shed light on central biological processes such as bacterial infection and memory, and drive advances in nanotechnology and materials science. In addition, amyloids play a key role in the wider pathways of protein folding, misfolding, and proteostasis, and studies of their structure and formation are essential for our fundamental understanding of these processes.

Amyloids are insoluble and non-crystallizable, so their structures have historically been challenging to study. Nonetheless, recent advances have allowed high-resolution structures to be obtained. While early work using X-ray fiber diffraction gave the first indications of cross-β structure (Astbury and Street, 1935; Eanes and Glenner, 1968; Blake and Serpell, 1996), the first detailed structural models were provided by solid-state nuclear magnetic resonance (ssNMR) spectroscopy studies of amyloids assembled *in vitro* from peptide fragments (Benzinger et al., 1998; Balbach et al., 2000; Jaroniec et al., 2002) or full-length polypeptides (Antzutkin et al., 2000; Balbach et al., 2002; Petkova et al., 2002; Heise et al., 2005; Lührs et al., 2005; Shewmaker et al., 2006; Paravastu et al., 2008), and X-ray crystallography of amyloid-like peptide microcrystals (Balbirnie et al., 2001; Nelson et al., 2005; Sawaya et al., 2007). These early studies emphasized the role of key interactions such as π-stacking, amide ladders, and salt bridges in stabilizing the cross-β structure (Balbirnie et al., 2001; Gazit, 2002; Petkova et al., 2002; Makin et al., 2005; Nelson et al., 2005), provided crucial information on molecular packing within the fibril core (Nelson et al., 2005; Sawaya et al., 2007), and demonstrated the diversity of amyloid structures, including the existence of polymorphism, where a single polypeptide chain can give rise to multiple distinct amyloid structures (Heise et al., 2005; Paravastu et al., 2008). In addition, atomic force microscopy (AFM), electron microscopy (EM), and early cryogenic electron microscopy (cryo-EM) studies gave insights into the molecular organization and mesoscale properties of amyloid fibrils, including their chirality, flexibility, and tensile strength, and provided further evidence for widespread polymorphism (e.g., Jiménez et al., 2002; Knowles et al., 2006, 2007; Smith et al., 2006; Meinhardt et al., 2009; Xue et al., 2009). In the last 5 years, the cryo-EM revolution has led to an explosion of high-resolution fibril structures, revealing a plethora of hitherto unforeseen features and shedding new light on the molecular basis of amyloid self-assembly; readers are referred to the reviews by Iadanza et al. (2018a), Ragonis-Bachar and Landau (2021), and Zielinski et al. (2021) for a summary of these recent advances. At the same time, the development of techniques for seeding or extraction of amyloid fibrils from tissue samples has allowed structural comparison of fibrils produced *in vitro* to those derived *ex vivo*, and has revealed the importance of the physiological environment in determining fibril structure, and the close association between polymorphism and disease phenotype (Fitzpatrick et al., 2017; Qiang et al., 2017; Kollmer et al., 2019; Schmidt et al., 2019; Zhang et al., 2019; Schweighauser et al., 2020; Bansal et al., 2021; Yang et al., 2022). Advances in AFM methodology, such as the development of tip deconvolution techniques, have also extended the resolution of AFM and allowed large-scale surveying of amyloid fibril polymorphism in near-atomic detail (Aubrey et al., 2020; Lutter et al., 2020). In this review, we take advantage of this rapid expansion of high-resolution molecular information to perform a broad comparison of the structures of amyloid fibrils formed by diverse experimental systems, including synthetic peptides, recombinant polypeptides induced to assemble *in vitro*, and amyloids seeded or extracted *ex vivo*. In particular, we examine the shared features of these structures, and highlight the underlying principles that give rise to them. Despite system-specific differences, we observe a high degree of commonality. We argue that the recurring features of amyloids point to general principles that govern their structure and activity, and are ultimately attributable to the unique geometry of the cross-β structure. In turn, these principles may help to explain why different amyloids can perform either functional or pathogenic roles, and suggest broad strategies with which to inhibit or control amyloid structure and self-assembly.

## 2. Hierarchical Organization of Amyloid Fibrils

**Figure 2:**
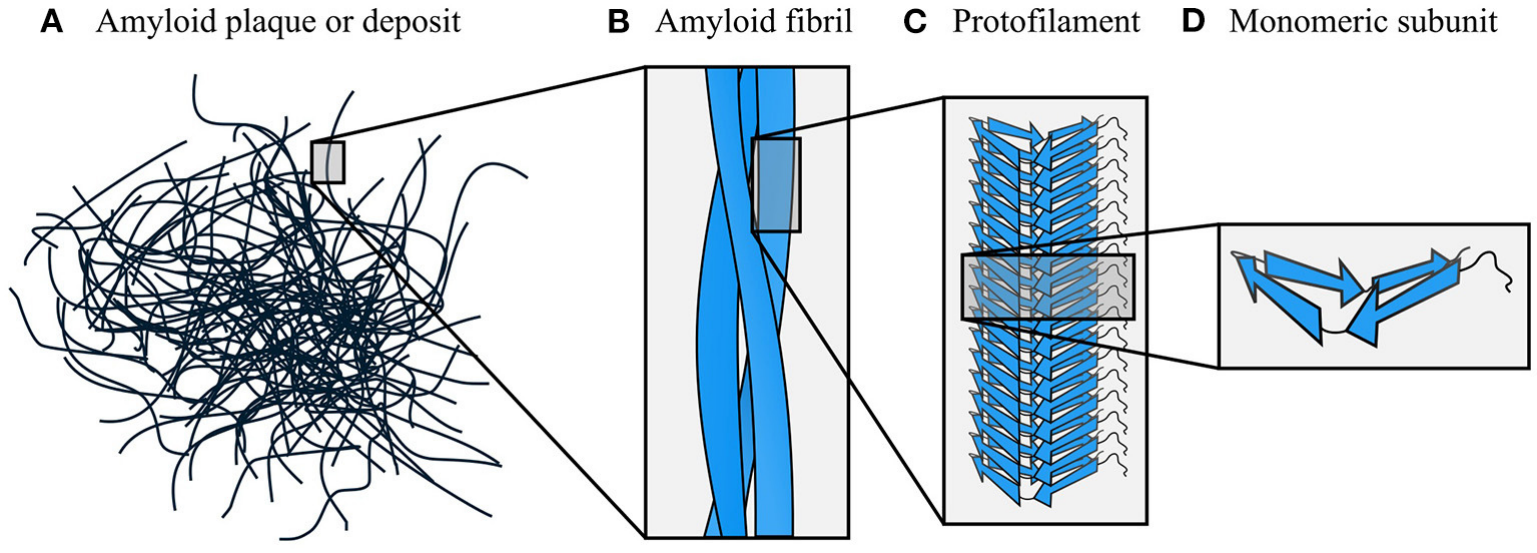
Hierarchical organization of amyloid fibrils. The dominant components of amyloid plaques or deposits are amyloid fibrils **(A,B)**, which are formed by close lateral association of protofilaments **(C)**. In turn, protofilaments are formed by stacking of monomeric subunits, and usually consist of a single stack **(D)**. The density map obtained by Mizuno et al. (2011) was used as a template for the fibril schematic in part **(B)**.

Amyloids have a hierarchical structural organization, consisting of symmetric associations of structural units formed at multiple different length scales (Figure 2). The terminology used to describe different levels of amyloids' hierarchical organization is not always consistent across the field; in this review, we have attempted to use the terms that are most neutral and least likely to cause confusion. Plaques or deposits of amyloid are composed of fibrous assemblies termed *fibers* or *fibrils*, although *fiber* can have other meanings and *fibril* is the most common term in structural studies (Figures 2A,B). A fibril consists of one or more laterally associated *protofilaments*, each of which is a long, filamentous assembly with its own continuous cross-β structure (Figure 2C). The protofilaments adhere tightly to one another with a well-defined symmetry and set of inter-protofilament packing interactions, and often wrap around one another to form a fibril with an overall twisted ribbon or helical morphology. In turn, each protofilament is a β-sheet hydrogen-bonded stack of monomeric *subunits* (Figure 2D). In some instances, protofilaments have been described as consisting of several laterally associated stacks of monomers, rather than a single stack (e.g., Paravastu et al., 2008). However, in most cases these structures can be reanalyzed as in-register associations of several separate protofilaments, each of which consists of a single stack of monomers (e.g., Bertini et al., 2011). In support of the latter interpretation, we note that attractive interactions between monomers are typically much stronger along the fibril axis than orthogonal to it (see Section 5), meaning that separate stacks of monomers only usually adhere to one another because their length permits a large number of mutually supporting interactions. As a result, subunit stacking is arguably situated at a more fundamental level of the organizational hierarchy than lateral association, and fibrils often exhibit polymorphism resulting from having a varying number or relative orientation of protofilaments, despite the protofilaments involved having similar monomer structures (e.g., Li et al., 2018a; Boyer et al., 2019). Therefore, in this review we mostly favor the interpretation where each protofilament consists of a single stack of monomeric subunits, unless there is a clear reason why a lateral grouping of monomers should adhere more strongly to one another than their neighbors along the fibril axis. Lastly, we note that some studies use the term proto*fibril* in place of proto*filament*; however, that particular usage is less common and is avoided in this review, as *protofibril* is also separately used to refer to entire, metastable, fibril-like structures distinct from mature amyloid fibrils (Walsh et al., 1997).

The hierarchical structure of amyloid fibrils means that different interaction motifs predominate in subunit stacking (Section 3), maintenance of a compactly folded protofilament structure (Section 4), and lateral association of protofilaments to form complete amyloid fibrils (Section 5). Throughout this review, we explore the extent to which this hierarchical differentiation is caused by the unique geometry of the cross-β structure, and the ways in which amyloids' structural organization and associated interaction motifs influence the functional, mechanical, and pathogenic properties of amyloid fibrils. It is important to note that, although we consider stacking, maintenance of a compact fold, and supra-protofilament assembly in separate sections, this simply reflects the fact that different symmetries and interaction motifs predominate at each of these organizational levels in mature amyloid fibrils. This separation does not necessarily entail a sequential development of structure in that particular order, and, in particular, given the high degree of cooperativity between interactions involved in stacking and subunit folding (Sections 3–4), it is likely that those two aspects of amyloids' structural organization develop at least partly in concert. Although, at several points in this review, we note possible implications for the dynamic process of amyloid formation, our primary aim is to consider the structural characteristics of mature amyloid fibrils, their likely causes, and the ways they may affect activity. Therefore, except where explicitly stated otherwise, arguments made in the following sections regarding the causes of amyloid formation should be understood to concern the thermodynamic driving factors, rather than the formation mechanism. Although, as with any cooperative process, higher-order organizational features such as supra-protofilament assembly help to stabilize more fundamental features such as subunit stacking, in balance, we argue that it is the unique geometry of the cross-β structure that is predominantly responsible for the other structural features that are widespread among amyloids, and their shared functional and pathogenic properties.

## 3. Stacking of Subunits to Form Protofilaments

**Figure 3:**
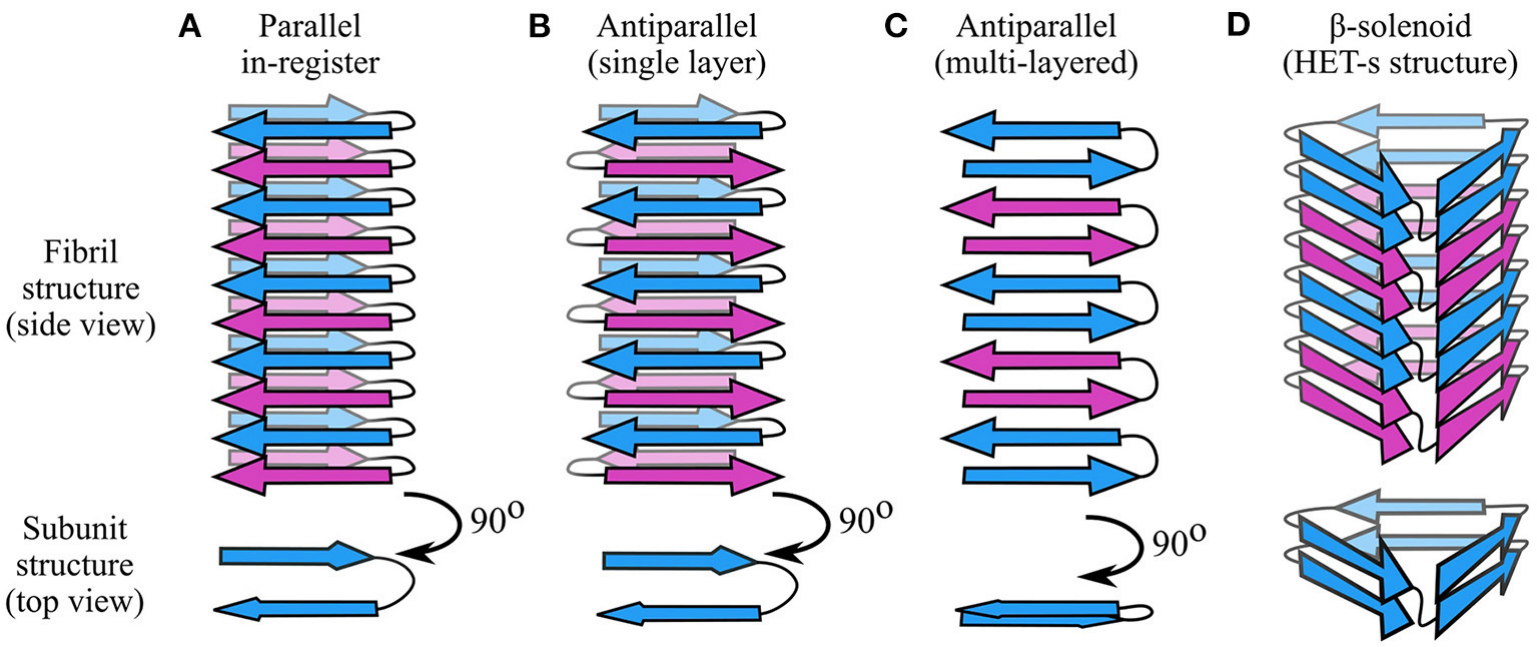
Types of cross-β structures. **(A)** In parallel in-register structures, each subunit contributes a single strand per intermolecular β-sheet, and the strands are oriented parallel and in-register with one another. Thus, the hairpin-like structure shown in this figure has two intermolecular β-sheets. **(B)** In single-layered antiparallel cross-β structures, each subunit contributes a single β-strand per β-sheet, but the strand direction alternates. **(C)** In multi-layered antiparallel structures, each subunit contributes more than one strand per β-sheet. **(D)** In β-solenoids such as HET-s (Wasmer et al., 2008), subunits occupy more than one layer by coiling in a solenoidal fashion. In these schematics, adjacent subunits are alternately colored blue and purple. Each monomeric subunit in the parallel in-register and antiparallel structures is a two-strand hairpin, differing only in orientation of the strands; a different monomer structure is used for the β-solenoid, based on a simplification of the HET-s structure (Wasmer et al., 2008).

As described in the previous section, each protofilament consists of a stack of monomeric subunits that collectively form a cross-β structure. Although there are notable exceptions (e.g., Wasmer et al., 2008; Vázquez-Fernández et al., 2016; Ghosh et al., 2021), the subunits usually have a flattened, single-layered tertiary structure containing one or more β-strands with the backbone hydrogen bonding groups oriented parallel to the protofilament axis (Figure 2D). As a result, the protofilament as a whole contains one or more intermolecular β-sheets, with each subunit contributing a single β-strand per β-sheet. Adjacent subunits may have peptide backbones oriented parallel or antiparallel to one another, giving rise to parallel or antiparallel cross-β structures (Figure 3), although the former type is more commonly observed. In this section, we provide an overview of the dominant forces and structural principles that drive subunit stacking, consider the conflicting factors that lead to formation of parallel or antiparallel cross-β structure, and discuss how the coordinated alignment of backbone hydrogen bonding groups along a shared axis predisposes amyloids to distinctive interaction motifs, such as steric zippers and amide ladders.

### 3.1. Dominant Forces in Subunit Stacking

**Figure 4:**
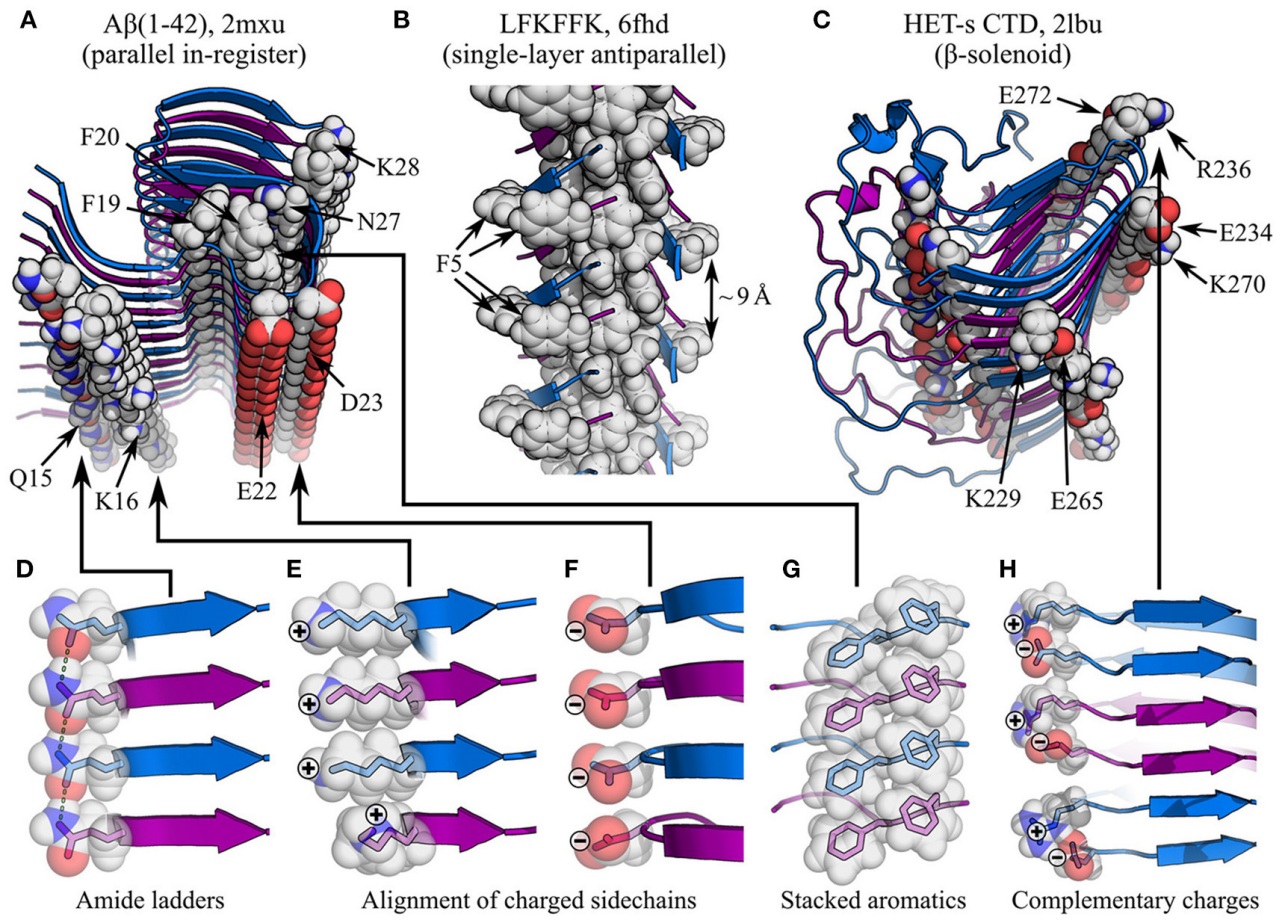
Impact of sidechain interactions on subunit stacking. **(A–C)** show of segments of amyloid fibrils with different types of cross-β structure, with specific interactions highlighted: **(A)** parallel in-register Aβ(1-42) fibrils (Xiao et al., 2015), showing amide ladders (Q15 and N27), alignment of charged sidechains (K16, E22, D23, and K28), and π-stacking (F19 and F20); **(B)** antiparallel LFKFFK fibrils (Salinas et al., 2018), showing a π-stacked core, but sub-optimal spacing of aromatic rings around the periphery (F5); **(C)** the β-solenoidal HET-s CTD (Wasmer et al., 2008), showing alignment of complementarily charged sidechains (K229-E265, E234-K270, and R236-E272) and amide ladders (unlabelled). The name of the polypeptide is given above each structure, alongside the PDB ID. Structures are shown as ribbon diagrams, with adjacent subunits alternately colored blue and purple for discrimination. Sidechains of interest are highlighted as spheres, with the color scheme: gray, carbon/hydrogen; red, oxygen; blue, nitrogen. **(D–H)** show close-up views of specific interactions in **(A–C)**, with semi-transparent rendering of the spheres to show the carbon/oxygen/nitrogen bonding structure within. In **(D)**, sidechain-sidechain hydrogen bonds are highlighted as green dashed lines. Note that the favorable stacking of aromatics in **(A,G)** contrasts with the suboptimal spacing between F5 rings in **(B)**, although the sequence degeneracy of LFKFFK means it is still possible to form stacks of aromatics within the fibril core.

Interactions between monomers along the protofilament axis are clearly dominated by backbone hydrogen bonding (Fändrich et al., 2001; Knowles et al., 2007; Fitzpatrick et al., 2011); although the hydrophobic effect and van der Waals forces play an important role in subunit folding and interactions between protofilaments, their role in stacking itself is much more limited. This means that the balance of interactions that defines the topology of amyloid fibrils is different from that observed in globular proteins, where the hydrophobic effect plays a more prominent role in maintaining a globular structure (Dill, 1990; Fitzpatrick et al., 2011). Accordingly, the anisotropic nature of backbone hydrogen bonding is responsible for the extreme aspect ratio of amyloid fibrils, in contrast to globular proteins whose folding is dominated by more isotropic forces (Knowles et al., 2007; Fitzpatrick et al., 2011). As will be outlined in this review, the significant topological differences between amyloids and globular proteins, particularly the scale, uniformity, anisotropy, and repetitiveness of the former, affect the nature of supplementary molecular interactions. At the level of subunit stacking, this is particularly pronounced in parallel in-register cross-β structures, where the alignment of the same amino acids in stacked subunits induces the formation of massive arrays of mutually polarized amide sidechains and π-stacked aromatics (Perutz et al., 1994; Gazit, 2002; Makin et al., 2005; Nelson et al., 2005; Tsemekhman et al., 2007; Figure 4), which complement the geometry and extensibility of the cross-β structure. At the same time, stacking of charged residues results in an unfavorable enthalpic contribution that opposes this alignment (Trovato et al., 2006; Figure 4). The global shift in the balance of interactions, from a situation dominated by hydrophobic collapse to one dominated by hydrogen bonding, has further important implications for self-assembly and activity. Due to the open-endedness of backbone hydrogen bonding and other interactions involved in subunit stacking, amyloids are infinitely extensible along a single axis, allowing them to self-replicate by templated structural conversion at their ends (Jarrett and Lansbury, 1993). As will be discussed in Sections 4 and 5, the repetitive organization of amyloids favors flattened subunit structures that facilitate stacking, and allows the formation of zipper-like interaction motifs that play a crucial role in stabilizing the subunit fold and promoting inter-protofilament interactions. In addition, the repetitive organization of the protofilament and the flattened structure of subunits create surface features such as exposed hydrophobics at the fibril ends, solvated channels, and strips of solvent-exposed functional groups, which may be responsible for activities such as Congo red and ThT binding (Wu et al., 2007, 2008, 2009; Biancalana et al., 2009), secondary nucleation (Barz and Strodel, 2016), and membrane disruption (Xue et al., 2009; Milanesi et al., 2012; Kollmer et al., 2016).

### 3.2. Entropic Considerations

While the high degree of structural order exhibited by amyloids would be expected to result in an unfavorable entropy of formation, concomitant burial of hydrophobics is also associated with a favorable desolvation entropy, which partly mitigates these losses just as it does for globular proteins. In addition, desolvation creates a less dielectric environment within the fibril, strengthening hydrogen bonding in the cross-β core (Nelson et al., 2005). Existing structures suggest at least two stages of assembly at which desolvation is likely to occur: firstly, during folding of the subunits, whether this happens before or during their assembly into a protofilament, and, secondly, when forming a dry interface between laterally associated protofilaments. It is also worth noting that most amyloids retain large disordered regions around their periphery, and domains that are well-folded in the native state may become less ordered in the amyloid. For example, while the N-terminal domain (NTD) of the yeast prion HET-s is folded in the non-amyloid state, it is a molten globule in the amyloid (Wasmer et al., 2009); on the one hand, this may help to mitigate the loss of chain entropy in the cross-β core, whereas on the other hand the loss of structure is likely to be accompanied by an unfavorable interaction enthalpy and solvation entropy. Monomer rigidity also strongly affects amyloid formation. More flexible polypeptides suffer from a greater loss of chain entropy during cross-β structure formation; as a result, under physiological conditions, chains with a low glycine content tend to aggregate to form amyloids, while those with a high glycine content tend to remain as solvated, disordered elastomers, despite being in an aggregated state (Rauscher et al., 2006).

### 3.3. Parallel Cross-β Structures

As previously discussed, the subunits of a protofilament can assemble to form a parallel or antiparallel cross-β structure (Figures 3A–C, 4A,B). While antiparallel structures have variable registry between the stacked β-strands, parallel structures almost always have an in-register alignment, meaning that identical residues are positioned on top of one another, with the sequences exactly aligned. This implies that the forces responsible for stabilizing parallel orientations are strongly dependent on the alignment of identical sidechains. An obvious candidate for such an interaction is π-stacking, which would be expected to occur along the extensive ladders of aromatic residues formed both within and on the exterior of amyloid fibrils (Gazit, 2002; Makin et al., 2005; Nelson et al., 2005; Figures 4A,G), in a manner similar to the stacking of nucleobases within nucleic acids. The importance of π-stacking is confirmed by existing ssNMR and cryo-EM structures (e.g., Madine et al., 2008; Fitzpatrick et al., 2017; Iadanza et al., 2018b; Liberta et al., 2019; Schmidt et al., 2019). Parallel in-register alignment is also stabilized by amide ladders, formed by hydrogen bonding between the aligned amide sidechains of stacked subunits (Figures 4A,D). Amide ladders were first identified in polyglutamine (Perutz et al., 1994), and have since been discovered in amyloid structures obtained by a wide variety of techniques (e.g., Chan et al., 2005; Nelson et al., 2005; Wasmer et al., 2008; Tuttle et al., 2016; Wälti et al., 2016; Fitzpatrick et al., 2017; Glynn et al., 2020; Röder et al., 2020). Molecular simulations have revealed that both sidechain and backbone amides form unusually strong hydrogen bonds between stacked subunits, due to polarization of their electron density by interactions with amides above and below them in the stack, and the resulting collective enhancement of the dipoles of aligned amides along the length of the protofilament. This effect is cooperatively reinforced as stack size increases, so that longer protofilaments have a more negative free energy per amide, and the hydrogen bonds involved in subunit stacking can be more stable than those found in crystalline ice (Tsemekhman et al., 2007). While this effect applies to both backbone and sidechain hydrogen bonding, it adds to the thermodynamic advantage of parallel in-register structures that are able to form amide ladders. In addition, the self-stabilizing nature of hydrogen bonding creates a non-linear dependence of the free energy of protofilaments on their length, which may partly explain the nucleation barrier for amyloid fibril formation (Tsemekhman et al., 2007).

**Figure 5:**
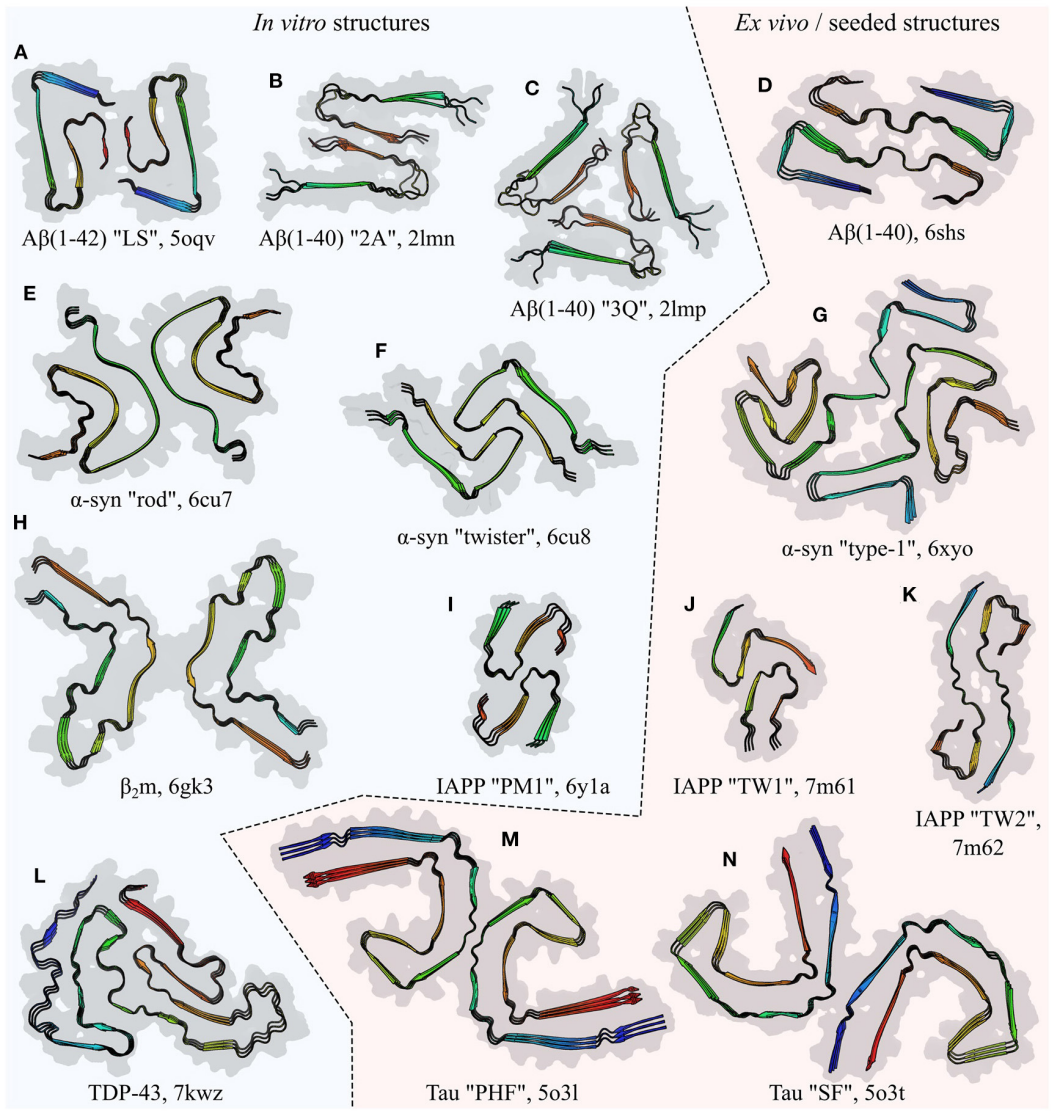
Comparison of the folds of amyloid fibril subunits, illustrated by examples from six polypeptides: amyloid-β (Aβ; Paravastu et al., 2008; Gremer et al., 2017; Kollmer et al., 2019), α-synuclein (α-syn; Li et al., 2018a; Schweighauser et al., 2020), β_2_-microglobulin (β_2_m; Iadanza et al., 2018b), islet amyloid polypeptide (IAPP; Röder et al., 2020; Cao et al., 2021), TDP-43 (Li et al., 2021), and Tau (Fitzpatrick et al., 2017). The name of the polypeptide is given below each structure, alongside the polymorph in quotes where relevant, and the PDB ID. Each structure is a stack of three subunit layers, viewed from a perspective facing down the fibril axis and using the same scale for all panels. Structures are composites of the surface (gray) and ribbon diagram (colored) representations, with the color of the latter varying spectrally from the N-terminus (blue) to the C-terminus (red); the true termini are used for spectral coloring of **(A–K)**, whereas the ends of the structured segments are used for **(L–N)**. Unstructured segments are not shown. Fibrils produced entirely *in vitro* are shown on the left of the central dashed line, while those extracted **(D,G,M,N)** or seeded **(J,K)** from living tissue are shown on the right.

It should also be noted that parallel in-register structures facilitate coordinated, thermodynamically advantageous folding of subunits, which is more difficult for antiparallel structures whose sequences are not aligned. It is now understood that the majority of amyloids formed by longer polypeptide chains have a highly complex tertiary structure, containing multiple β-strands interspersed with turns and disordered segments (e.g., Tuttle et al., 2016; Fitzpatrick et al., 2017; Gremer et al., 2017; Liberta et al., 2019; Schmidt et al., 2019; Swuec et al., 2019; Röder et al., 2020; Li et al., 2021; Yang et al., 2022; see Figure 5 for examples, and Section 4 for more detail). Compared to a simpler tertiary structure, a more complex structure is often better able to maximize favorable interactions and avoid unfavorable interactions, resulting in greater stability. However, if the chain direction were to alternate between subunits, the differing distribution of residues such as prolines and glycines, which affect the distribution of turns and β-strands, would make it difficult for layered subunits to have β-strands in the same place. Thus, longer polypeptides that prefer to form multiple β-strands are more likely to successfully find a stable, folded subunit structure if they adopt a parallel in-register alignment.

### 3.4. Antiparallel Cross-β Structures

The primary effect that disfavors parallel in-register structures, and favors antiparallel structures, appears to be the electrostatics (Figures 4A,E,F). Alignment of the termini and charged sidechains of parallel in-register subunits results in an unfavorable enthalpic term, which can be lessened by adopting an antiparallel arrangement (Trovato et al., 2006). Accordingly, one would expect polypeptides with a higher content of aromatics and sidechain amides to prefer a parallel in-register alignment, while those with more charged sidechains would prefer an antiparallel alignment. There is also a length effect, since shorter chains are likely to have a higher fraction of charged residues due to the length-independent charges at their termini, and, as discussed in Section 3.3, shorter chains also experience less pressure to align amino acids such as glycine and proline, which influence the position of turns and β-sheets in the subunit structure. It is also worth noting that the improved hydrogen bonding geometry of antiparallel β-sheets may favor antiparallel cross-β structures, although this effect is likely to be small. In most cases, the factors favoring parallel alignment appear to win out, but there are occasional instances where antiparallel cross-β structures appear to be stable; these include the sequence-designed peptide KFFEAAAKKFFE (Makin et al., 2005), a polymorph of the LFKFFK fragment of the cytotoxic PSMα3 peptide from *Staphylococcus aureus* (Salinas et al., 2018; Figure 4B), the small Aβ-derived peptides Aβ(11–25) (Petkova et al., 2004) and Ac-KLVFFAE-NH_2_ (Balbach et al., 2000; Bu et al., 2007), and a recent structural model of Huntingtin exon 1 (HttEx1) (Boatz et al., 2020). In the above cases, antiparallel alignment appears to be attributable to either the shortness of the peptide (KFFEAAAKKFFE, LFKFFK, and the Aβ-derived peptides), or the complementarity or degeneracy of the primary sequence (KFFEAAAKKFFE and HttEx1), which allows π-stacking and amide ladders to occur in a similar manner to parallel in-register structures.

In general, antiparallel cross-β structure is rare in amyloids formed by longer polypeptides with nondegenerate sequences, and, where exceptions to do occur, the resulting fibrils tend to be metastable. For example, a polymorph formed by the Iowa mutant (D23N) of Aβ(1–40) had a single-layered antiparallel structure similar to that shown in Figure 3B, but these fibrils were metastable and were ultimately replaced by parallel in-register fibrils (Qiang et al., 2012). In addition, antiparallel β-sheets are often observed in metastable oligomers and filamentous species formed transiently during amyloid formation (Yu et al., 2009; Sandberg et al., 2010; Dupuis et al., 2011; Sarroukh et al., 2011; Laganowsky et al., 2012). Direct or indirect conversion from antiparallel to parallel β-sheets has been suggested to be a slow step in fibril nucleation or maturation (Sandberg et al., 2010; Qiang et al., 2012), and antiparallel oligomers and fibrils are often found to be toxic (Sandberg et al., 2010; Laganowsky et al., 2012; Liu et al., 2012; Qiang et al., 2012), suggesting that factors that prolong the lifetime of such assemblies may have a major impact on pathology. Lastly, we note that a recent cryo-EM density map of Aβ(1–40) fibrils (Ghosh et al., 2021), seeded with patient-derived material from Alzheimer's disease cortical tissue, had a parallel in-register core (as in Figure 3A) surrounded by peripheral density that was most consistent with two additional protofilament-like stacks of monomers in an intramolecular β-hairpin conformation (as in Figure 3C). This resulted in an overall fibril with a combination of parallel and antiparallel cross-β structure. In this instance, the fibrils appeared to be stable for long timescales, although this may well have been the due to the stabilizing effect of the parallel in-register core, which ssNMR data indicated was more ordered.

### 3.5. Solenoidal Cross-β Structures

In the majority of protofilament structures, each subunit consists of a single layer of β-strands and other secondary structural elements. The main reason for this may be the comparative stability of parallel in-register motifs; these are most easily formed if each subunit contributes only a single β-strand per intermolecular β-sheet, favoring quasi-planar subunits that are flattened in the plane orthogonal to the protofilament axis. Nonetheless, there is a major exception to this rule, in the form of amyloids consisting of stacked β-solenoid subunits (Figures 3D, 4C). In these structures, each subunit folds along the protofilament axis to form a multi-layered solenoid; the prototypical example of this is the C-terminal domain (CTD) of the HET-s prion from the fungus *Podospora anserina*, in which the polypeptide chain folds upon itself in a left-handed β-helical manner to form a two-layered structure with three parallel β-sheets, each consisting of a pair of stacked β-strands. Subunit stacking then assembles these sheets into a cross-β protofilament with a β-solenoid structure, in which each subunit contributes two aligned β-strands to each of the structure's three parallel intermolecular β-sheets (Wasmer et al., 2008; Figure 3D). The HET-s CTD structure is remarkable for the elegant manner in which it resolves the conflicting requirements to form favorable in-register interactions and avoid electrostatic repulsion between aligned charges (Figure 4C). As would be expected, the two layers of the β-helix have a high degree of sequence complementarity to achieve this. Although π-stacking interactions are not present in the cross-β core, there are two amide ladders formed by the residue pairs N226-N262 and N243-N279, which run along the protofilament in an alternating fashion (Figure 4C). However, by adopting a two-layered pseudo-in-register alignment, the HET-s CTD is also able to avoid unfavorable alignment of like charges between stacked β-strands; instead, there is a system of complementary alternating charges created by the residue pairs K229-E265, E234-K270, and R236-E272 (Wasmer et al., 2008, 2009; Figures 4C,H). Besides the obvious enthalpic advantages of this structure, it is worth noting that folding of the β-solenoidal subunit is based on more local interactions than in most amyloids, where interactions between separate subunits are likely to be required for the final tertiary structure to appear. This may encourage rapid folding and emergence of a mature subunit structure prior to assembly, potentially explaining the apparent lack of evidence for non-fibrillar intermediates formed by HET-s. Given their functional role, and the toxic effects of many amyloid-related oligomers, there is a clear incentive for yeast prions to form via a predominantly two-state process, and the concentrations of intermediates formed by the Ure2p prion have previously been shown to be low compared to other amyloids (Dear et al., 2020). By adopting a β-solenoid subunit structure, HET-s may thus be able to avoid primary nucleation intermediates altogether. However, it is worth noting that toxic species can also be formed by other processes, such as fragmentation (Xue et al., 2009) and fibril-mediated secondary nucleation (Ruschak and Miranker, 2007; Cohen et al., 2013; Frankel et al., 2019), and functional amyloids might also require adaptations to limit these risks. Besides HET-s, β-solenoids have now been induced in engineered amyloids based on modifications of existing β-solenoid proteins (Peralta et al., 2015; Peng et al., 2020), and there are data to suggest that at least one polymorph of the mammalian prion protein may have a four-layered β-solenoid structure (Vázquez-Fernández et al., 2016).

## 4. Tertiary and Quaternary Structure of Protofilaments

In the majority of amyloids, the subunits are single-layered or rarely multi-layered monomers that fold to produce convoluted but flattened tertiary structures (Figure 5). At the same time, subunit stacking forms the quaternary structure of the entire protofilament, and provides crucial contacts that stabilize the tertiary structure, making the two highly interdependent. While early models of amyloids had relatively simple subunit structures organized from a small number of secondary structural elements, such as the β-hairpin-like models of Aβ(1–40) (Petkova et al., 2002) and Aβ(1–42) (Lührs et al., 2005), it has since become clear that many amyloids have subunits containing a large number of separate turns and β-strands, with a complex tertiary organization. In some of the more complex structures, this has been likened to a Greek key (Tuttle et al., 2016), although the term “amyloid key” (Liberta et al., 2019) may better represent the unique characteristics of this motif, as the amyloid key differs from a canonical Greek key in having backbone hydrogen bonding groups oriented orthogonal, rather than parallel, to the plane of the motif, in order to form hydrogen bonds with adjacent monomers. Reports of differing levels of structural complexity for the same polypeptide are not mutually incompatible, since amyloids are often highly polymorphic; thus, in some cases the formation environment and the natural tendency of non-functional amyloids to nucleate a variety of different polymorphs may result in fibrils with a more or less complex tertiary structure. In addition, studies of amyloids such as Aβ (Xiao et al., 2015; Wälti et al., 2016; Gremer et al., 2017; Kollmer et al., 2019; Yang et al., 2022), α-syn (Tuttle et al., 2016; Guerrero-Ferreira et al., 2018, 2019; Li et al., 2018a,b; Boyer et al., 2019; Zhao et al., 2020), and IAPP (Röder et al., 2020; Cao et al., 2021) have revealed a high degree of polymorphism resulting from protofilaments having distinct but comparably complex structures.

### 4.1. Subunit Compaction and Desolvated Core Formation

**Figure 6:**
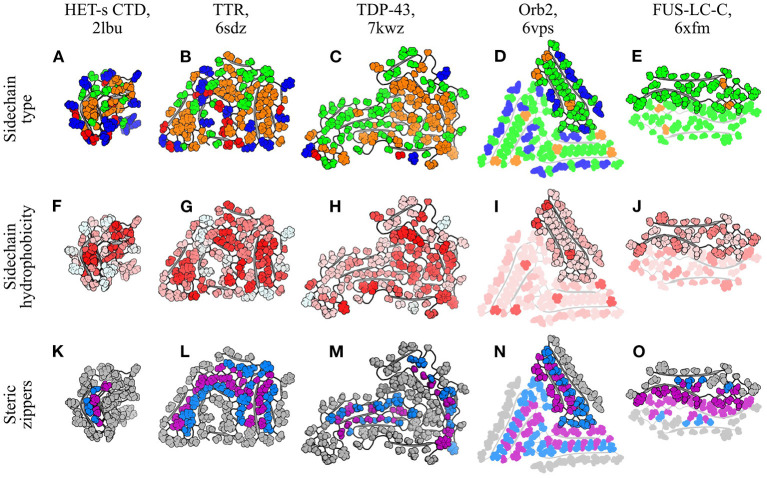
Segregation and packing of sidechains in amyloid fibrils. Five different fibril structures are shown, organized by column: **(A,F,K)**, HET-s CTD (Wasmer et al., 2008); **(B,G,L)**, TTR (Schmidt et al., 2019); **(C,H,M)**, TDP-43 (Li et al., 2021); **(D,I,N)**, Orb2 (Hervas et al., 2020); **(E,J,O)**, FUS-LC-C (Lee et al., 2020). The name of the polypeptide is given above each structure, alongside the PDB ID. Each structure is a single subunit layer, viewed from a perspective facing down the fibril axis and using the same scale for all panels. Structures are composites of the ribbon (gray) and spheres (colored) representations, with the latter used to specifically highlight sidechains. Three different color schemes are used for sidechains, with one per row. **(A–E)** are colored according to sidechain type: red, negatively charged (D/E); blue, positively charged (K/R); green, hydrophilic uncharged (Q/N/S/T/Y); orange, hydrophobic (A/C/F/I/L/M/P/V/W). Histidines are colored blue or green according to the expected protonation state. **(F–J)** are colored according a normalized hydrophobicity scale (Eisenberg et al., 1984), with the most hydrophobic residues colored red and the most hydrophilic colored white. **(K–O)** are colored to highlight selected steric zippers, with sidechains in zipper-forming strands alternately colored either blue or purple, so that the two halves of each intra-protofilament zipper are colored differently. For the purpose of this figure, a steric zipper is defined as a chain segment whose sidechains are buried in the fibril core and interdigitated between the sidechains of an opposing chain segment. This includes cases varying from a relatively low [e.g., **(K)**] to a high [e.g., **(N)**] level of interdigitation, and reflects the fact that steric zippers, as defined here, exist on a continuum rather than having a simply defined cut-off. For clarity, only some of the zipper segments have been highlighted for TDP-43. For the Orb2 and FUS-LC-C structures, which have multiple protofilaments, additional protofilaments are shown with semi-transparent rendering, to aid in discrimination between protofilaments and identification of inter-protofilament steric zippers. Note that FUS-LC-C contains an inter-protofilament homo-zipper, which is formed by identical chain segments from either side of the protofilament binding interface, and has the same color for each half of the zipper.

The fold of subunits is stabilized by two distinct sets of interactions. While interactions along the protofilament axis maintain stacking, interactions between chain segments orthogonal to that axis keep the subunits in a compact conformation, usually consisting of multiple turns and β-strands centered around a desolvated core (Figure 5). A compact subunit conformation is both entropically and enthalpically favored. An extended chain conformation is statistically improbable for all but the shortest amyloidogenic peptides, and adoption of a compact fold allows amyloids to minimize unfavorable interactions between hydrophobic chain segments and water, and maximize favorable interactions between complementary chain segments such as ladders of oppositely charged sidechains. While the hydrophobic effect and van der Waals forces play only a minor role in interactions along the protofilament axis, they are usually the dominant driver for compaction of the subunit orthogonal to that axis. The majority of single-layered subunit structures have a desolvated core containing clusters of hydrophobic residues, while the hydrophilics are typically, but not exclusively, exposed to the solvent (Figures 6A–J). For example, in the multi-layered β-solenoid of HET-s, the β-helix of the CTD has a hydrophobic cluster of residues at the center, with the hydroxyl and charged sidechains on the outside (Wasmer et al., 2008; Figures 6A,F). This orientation effect is usually particularly pronounced for charged sidechains, as well as the N- and C-termini in relevant cases, as charged groups experience a highly unfavorable free energy change upon transfer from the solvent to the less dielectric interior of the structure (Figures 6A–E). However, there is also a weaker preference for exposure of uncharged polar groups (Figures 6A–E), and the segregation of residues by hydrophobicity in amyloids broadly resembles the formation of a hydrophobic core in globular proteins (Figures 6F–J). More generally, it is also worth noting that the formation of a desolvated core strengthens backbone hydrogen bonding between β-sheets (Nelson et al., 2005), suggesting that desolvation may be coupled to either emergence or consolidation of the cross-β structure.

Inside the fibril core, complementary sidechains from adjacent chain segments typically interdigitate to form tight, zipper-like interfaces that exclude water and optimize van der Waals contacts (Figures 6K–O). These “steric zippers” were first identified in an inter-protofilament context in amyloid-like microcrystals of the GNNQQNY peptide (Nelson et al., 2005), but similar intra-protofilament interfaces have since been observed in many amyloid fibrils (e.g., Iadanza et al., 2018b; Cao et al., 2019; Schmidt et al., 2019; Hervas et al., 2020; Lee et al., 2020; Li et al., 2021). Hydrophobic sidechains are the most common constituents of intra-protofilament steric zippers; for example, the core of the HET-s CTD contains a small hydrophobic zipper (Wasmer et al., 2008; Figures 6A,F,K), and the structure of transthyretin (TTR) amyloid is maintained by several intra-protofilament hydrophobic zippers (Schmidt et al., 2019; Figures 6B,G,L). Nonetheless, hydrophilic sidechains can also form zippers, particularly in instances where sidechain-sidechain or sidechain-backbone hydrogen bonding partners are available to offset the loss of interactions with water. For example, intra-protofilament hydrophilic zippers have recently been observed in fibrils formed by TDP-43 (Li et al., 2021; Figures 6C,H,M), the memory-associated amyloid Orb2 (Hervas et al., 2020; Figures 6D,I,N), and a C-terminal segment of the FUS low-complexity domain (FUS-LC-C; Lee et al., 2020; Figures 6E,J,O). Steric zippers are remarkable for their high degree of regularity, and tight packing of sidechains in the subunit plane. Nonetheless, these unusual characteristics can still be explained in terms of the same principles that govern sidechain packing in globular proteins, subject to the distinct molecular symmetry of amyloid fibrils. While the regularity of steric zippers results from the repetitive structure of amyloids, the tight packing of sidechains in the subunit plane is consistent with the general principle that optimal packing densities are improved in a planar environment compared to a three-dimensional one (Torquato and Stillinger, 2006). Thus, steric zippers are arguably an inevitable consequence of sidechain packing and desolvated core formation in an assembly with a repetitive structure along a single axis, and a flattened subunit structure that enhances packing orthogonal to that axis. This may help to reconcile the importance of sequence-dependent effects on steric zipper formation with the near-universality of cross-β structure. On the one hand, some primary sequences are clearly more suitable for steric zipper formation than others, and this suitability results in a strong association between certain sequences and protein aggregation (Sawaya et al., 2007). On the other hand, amyloid formation is ultimately believed to be a near-universal property of polypeptide chains, accessible to almost all protein sequences under appropriate conditions (Chiti et al., 1999; Fändrich et al., 2001) and perhaps even the majority of “non-amyloidogenic” sequences under physiological conditions, meaning that kinetic trapping and the activity of chaperones are often the sole impediment to aggregation *in vivo* (Baldwin et al., 2011; Varela et al., 2018). The fact that such a wide range of protein sequences can form amyloid implies that specific zipper-forming sequences are not necessary for amyloid formation. Instead, steric zippers may be better viewed as an inevitable consequence of cross-β structure, which is still able to modulate the thermodynamics and kinetics of amyloid formation in a strongly sequence-dependent manner.

### 4.2. Polar Interactions in Subunit Folding

**Figure 7:**
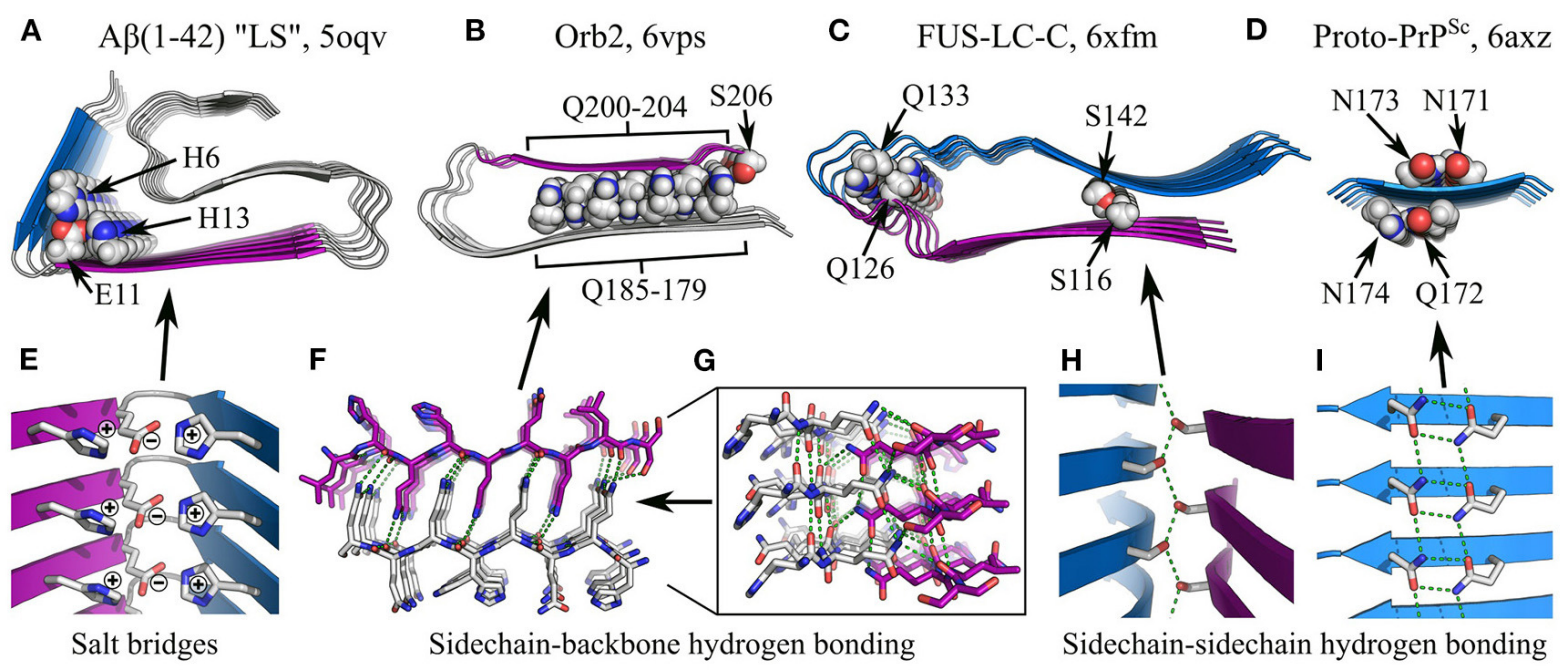
Specific interactions in subunit folding. **(A–D)** show the structures of various individual protofilaments, with specific interactions highlighted: **(A)**, Aβ(1-42) “LS” polymorph, (Gremer et al., 2017), showing the H6-E11-H13 salt bridge; **(B)**, Orb2 (Hervas et al., 2020), with sidechain-backbone hydrogen bonding by interdigitated glutamines, and sidechain-sidechain hydrogen bonding between Q179 and S206; **(C)**, FUS-LC-C (Lee et al., 2020), with sidechain-backbone hydrogen bonding from the sidechain amide of Q126 to the backbone carbonyl of Q133, and sidechain-sidechain hydrogen bonding between S116 and S142; **(D)**, amyloid-like microcrystals of the prion-derived proto-PrP^Sc^ peptide (Gallagher-Jones et al., 2018), showing intra-strand sidechain-sidechain hydrogen bonding (“polar clasps”) by the residue pairs N171-N173 and Q172-N174. The name of the polypeptide is given above each structure, alongside the polymorph in quotes where relevant, and the PDB ID. Structures are shown as ribbon diagrams, with chain segments colored gray, blue, or purple for discrimination. Sidechains of interest are highlighted as spheres, with the color scheme: gray, carbon/hydrogen; red, oxygen; blue, nitrogen. For **(B,C)**, missing hydrogens have been modeled in. Throughout panels **(A–D)**, all structures use the same scale. **(E–I)** show close-up views of specific interactions in **(A–D)**. In **(E,H,I)**, structures are shown as ribbon diagrams with sidechains as sticks, using the same color scheme as **(A–D)**; in **(F–G)**, both the backbone and sidechains are shown as sticks, with carbons in the Q200-S206 segment colored purple. For clarity, and consistency between structures with varying detail, hydrogens are not represented with sticks and are thus implicit. Hydrogen bonds are represented by green dashed lines.

In addition to the hydrophobic effect and van der Waals interactions, subunit folding can be maintained by specific polar interactions such as salt bridges and hydrogen bonding, both within and outside the context of steric zippers (Figure 7). While charged sidechains are typically solvent-exposed (Figures 6A–E), buried salt bridges often stabilize key turns in the subunit structure, such as the H6-E11 and E11-H13 salt bridges in the “LS” polymorph of Aβ(1–42) (Gremer et al., 2017; Figures 7A,E). Similarly, many subunit structures are stabilized by non-β-sheet hydrogen bonding interactions. For example, sidechain-backbone hydrogen bonding occurs in the protofilament core of Orb2, where glutamine repeats on either side of a dry intra-protofilament interface form an interdigitated system of amide ladders (Hervas et al., 2020; Figures 6D,I,N, 7B,F). In addition to backbone-backbone and sidechain-sidechain hydrogen bonds running along the protofilament axis, the -NH_2_ groups of the ladders each donate an additional hydrogen bond to the backbone carbonyls on the opposite side of the interface (Figure 7F), forming an extended tetragonal network of hydrogen bonds that holds the two β-sheets together (Figure 7G). It has been speculated that similar structures might be observed in amyloids formed by other glutamine-rich proteins, such as Huntingtin (Hervas et al., 2020). Besides this, Orb2 fibrils also contain intramolecular sidechain-sidechain hydrogen bonding between Q179 and S206 (Hervas et al., 2020; Figures 7B,F,G). In a similar manner, the fold of FUS-LC-C subunits is stabilized by sidechain-backbone hydrogen bonding from the sidechain amide of Q126 to the backbone amide of Q133, and sidechain-sidechain hydrogen bonding between S116 and S142 (Lee et al., 2020; Figures 7C,H), and microcrystals of the prion-derived proto-PrP^Sc^ peptide contain “polar clasps” formed by intra-strand hydrogen bonding between nearby amide ladders (Gallagher-Jones et al., 2018; Figures 7D,G). In a recent *ex vivo* polymorph of Aβ(1-42) amyloid (“type-I”), there was also a network of hydrogen bonds between the sidechains of E11, H13, and H14, which helped to stabilize the N-terminal region of the peptide (Yang et al., 2022). As with steric zippers, arrays of polar interactions form as a natural consequence of the repetitive structure of amyloids and the tendency of functional groups to segregate according to hydrophobicity during subunit folding. A notable feature of these motifs, as illustrated by the above examples, is that polar moieties can form specific, favorable interactions with several different partners at the same time; as a result, they often link up to form extensive, mutually supporting networks that further stabilize the protofilament structure (Figures 7G–I).

### 4.3. Non-planar Subunit Structures

**Figure 8:**
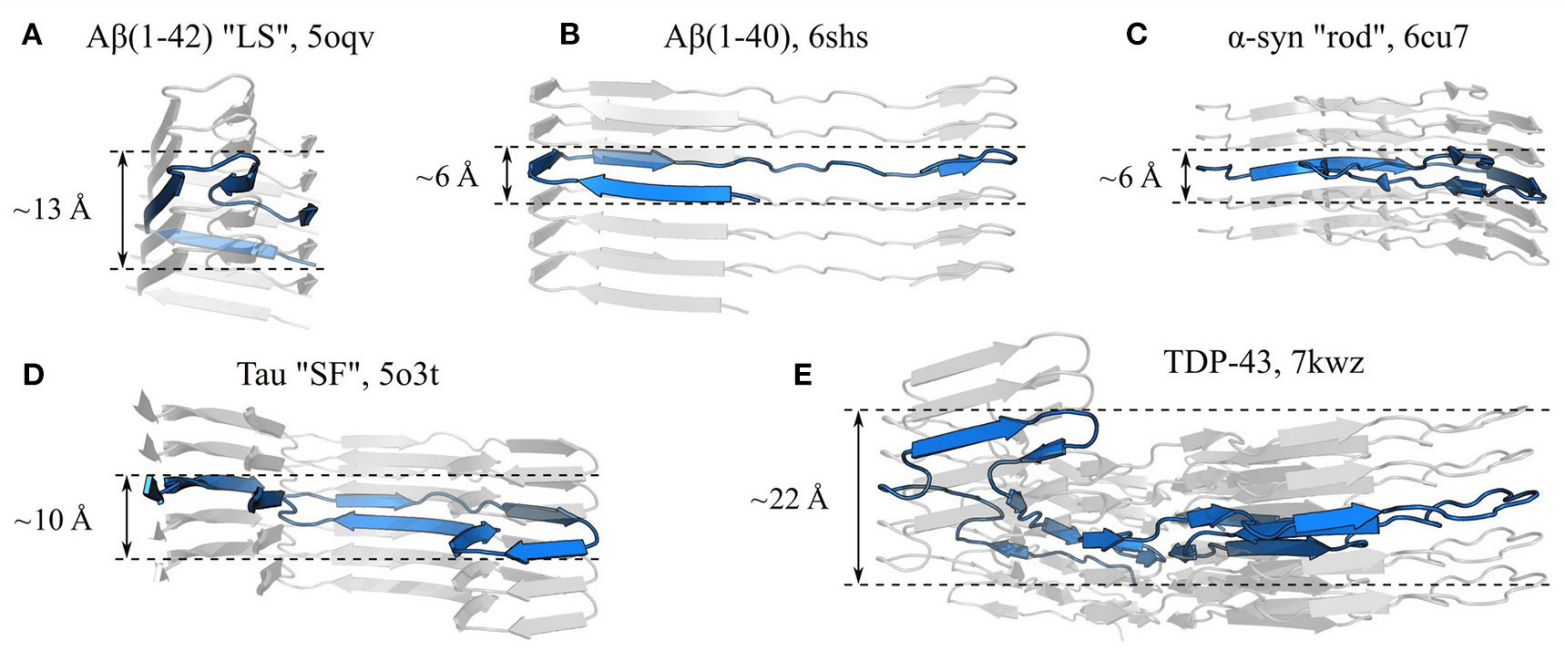
The subunits of amyloid fibrils often occupy a single layer of the protofilament stack, but are not truly planar. Panels show side-on views of single protofilaments from fibrils formed by **(A)** Aβ(1-42) “LS” polymorph (Gremer et al., 2017), **(B)** Aβ(1–40) (Kollmer et al., 2019), **(C)** α-syn “rod” polymorph (Li et al., 2018a), **(D)** Tau “SF” polymorph (Fitzpatrick et al., 2017), and **(E)** TDP-43 (Li et al., 2021), with a single subunit highlighted in blue in each case. The name of the polypeptide is given above each structure, alongside the polymorph in quotes where relevant, and the PDB ID. Measurements show the approximate subunit height variation along the protofilament axis. All panels use the same scale.

While subunits are most often single-layered structures, giving them a quasi-planar character, complete (or more accurately near-complete) planarity is actually quite rare; one of the best examples to date is the recent cryo-EM structure of a protease-resistant human prion fragment, PrP^*Sc*^(94–178), in which the coordinates of the C_α_ atoms of a single subunit vary by no more than ~3.6 Å along the protofilament axis (Glynn et al., 2020). Instead, the vast majority of structures have flexed, non-planar subunits, so that β-strands from the same subunit occur in different planes of the stack, and the orientation of the β-strands is only approximately orthogonal to the protofilament axis (e.g., Fitzpatrick et al., 2017; Gremer et al., 2017; Guerrero-Ferreira et al., 2018; Iadanza et al., 2018b; Röder et al., 2019, 2020; Hervas et al., 2020; Li et al., 2021; see Figure 8 for examples). This has three important implications for the structure and self-assembly of protofilaments. Firstly, the ability of a single subunit to simultaneously occupy different positions along the length of the protofilament means that interactions such as steric zippers and lateral hydrogen bonds can form between chain segments from different subunits in the stack, creating additional quaternary contacts that help to stabilize the structure. Secondly, subunits typically span a distance of more than one β-sheet spacing along the protofilament axis, allowing interactions between subunits that are not nearest neighbors in the β-sheet topology. These non-nearest-neighbor contacts create a more complex network of interactions between the subunits than a simple linear chain, which may help to cooperatively stabilize the protofilament structure, and explain why amyloid formation typically occurs as a nucleated phase transition, rather than a simple downhill self-assembly process. Thirdly, the non-planar subunit conformation adds to the existing polarity of the protofilament structure, and gives the fibril distinct, jagged binding interfaces for addition of new subunits at either end. While local variation in chain height enhances the overall jaggedness of the filament ends, global tilting, flexing, or spiraling of the subunits causes one end to have a different topography from the other. Consequently, many amyloids have distinct convex and concave surfaces at either end, which are sometimes termed “ridge” and “groove”, respectively (Gremer et al., 2017; Li et al., 2021), and these curved or “terraced” surfaces expose steric zipper segments and other motifs that would otherwise be confined to the fibril interior. An extreme example of this is seen in PI3K-SH3 fibrils, in which the total subunit height variation is ~15 Å, and this maximum height difference is achieved between chain segments that are positioned next to one another in the subunit topology, creating a deep binding pocket at one end of the fibril at which more than three stack layers are exposed, and a similarly conspicuous ridge at the other end (Röder et al., 2019). Other amyloids with pronounced “groove” and “ridge” ends include the Aβ(1–42) “LS” polymorph (Gremer et al., 2017), the α-syn “twister” polymorph (Li et al., 2018a), and fibrils formed by TDP-43 (Li et al., 2021), and most amyloids exhibit this phenomenon to some degree. The jaggedness, tilting, and curvature of the surface of the fibril ends will influence processes that occur there, such as elongation and lipid binding (Xue et al., 2009; Milanesi et al., 2012; Kollmer et al., 2016), and the distinct topography of the two ends helps to explain why association or dissociation of new monomers can be much faster at one end than at the other (Ban et al., 2004; Young et al., 2017).

### 4.4. Disordered Regions and Cofactors

Another important feature of the tertiary structure of subunits, which has been clear since early ssNMR studies (Balbach et al., 2002; Jaroniec et al., 2002; Heise et al., 2005; Lührs et al., 2005), is that only part of the polypeptide sequence typically contributes to the highly ordered cross-β core. The rest may exist in a disordered state (e.g., Fitzpatrick et al., 2017; Gremer et al., 2017; Guerrero-Ferreira et al., 2018; Iadanza et al., 2018b; Radamaker et al., 2019, 2021; Röder et al., 2020), or even as a relatively well-ordered surface domain often separated by a flexible linker region (e.g., Wasmer et al., 2009; Kryndushkin et al., 2011; Sivanandam et al., 2011). The decoration of the fibril surface with these non-amyloid domains would be expected to strongly affect surface-mediated processes such as supra-protofilament assembly, capture of monomers for elongation, production of toxic and/or fibrillogenic oligomers by fibril-mediated secondary nucleation, and binding of chaperones or disaggregating agents; for a more in-depth discussion of these effects, readers are referred to the recent review by Ulamec et al. (2020). At present, it is difficult to address the structural basis of these phenomena as prevailing techniques such as ssNMR and cryo-EM struggle to resolve the surface domains. For example, while additional density corresponding to these domains is often visible in cryo-EM density maps, local resolution is typically far too poor to model a polypeptide backbone. Areas of extra density are sometimes also interpreted as representing heterogeneous fibril constituents that stabilize the protofilament structure, or inter-protofilament interactions; in some cases these species may be metal ions and polyanions that help to balance aligned charges on the protofilament surface (e.g., Dearborn et al., 2016; Fitzpatrick et al., 2017; Yang et al., 2022), and some amyloids contain ordered water that participates in hydrogen bonding with nearby polar moieties (e.g., Gallagher-Jones et al., 2018; Lee et al., 2020).

## 5. Supra-Protofilament Assembly

**Figure 9:**
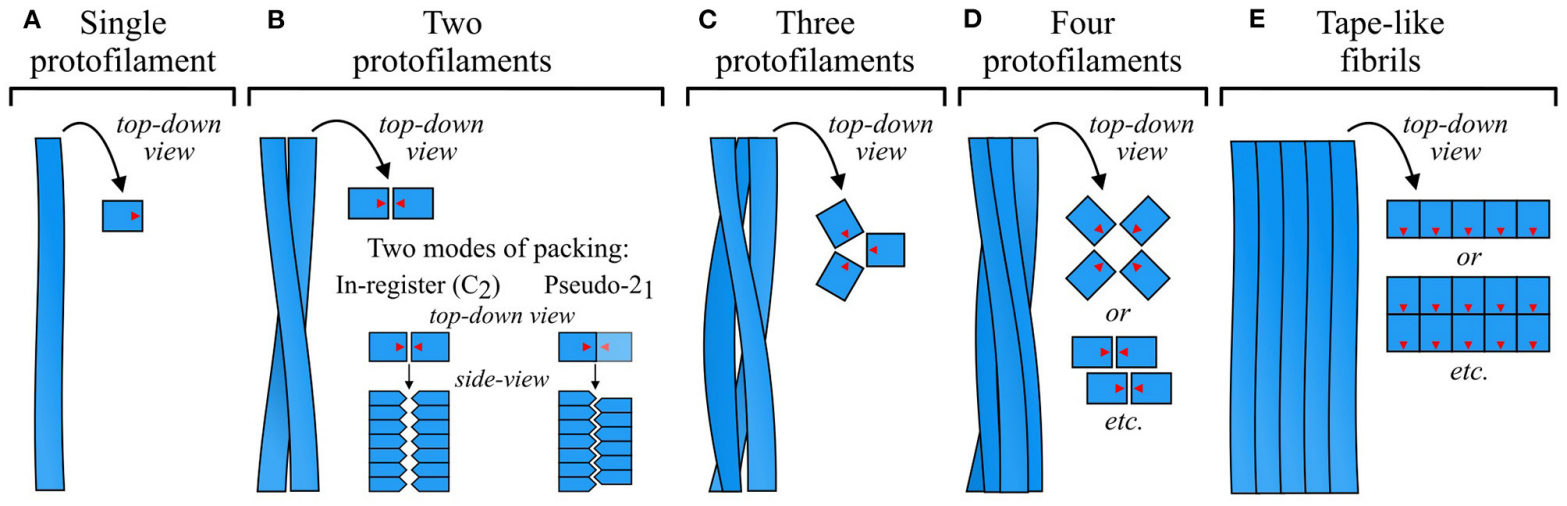
Schematic of common modes of protofilament organization. Fibrils can be **(A)** a single protofilament, **(B–D)** a twisted ribbon or tubular structure formed by association of several protofilaments, often with rotational symmetry about the fibril axis, or **(E)** a tape-like side-by-side association of protofilaments. In the top-down schematics, blue squares with red arrows represent top-down views of subunits, with the red arrows showing their relative orientation in the subunit plane. In the side-on schematics for **(B)**, blue pentagons represent side views of protofilament subunits. As shown in this panel, twofold-symmetric fibrils or protofilament groups can have an in-register (C_2_) association of laterally apposed subunit stacks, or a staggered (pseudo-2_1_) organization in which the two stacks are half a β-sheet spacing out of register. While the former optimizes interactions that rely on alignment of the subunits in the same plane, the latter allows interdigitation of sidechains that protrude into the interface. The density map obtained by Mizuno et al. (2011) was used as a template for the schematic in **(C)**.

In many cases, amyloids consist of associations of several protofilaments that wrap around one another to produce a complete fibril with a twisted ribbon or helical morphology (Figure 2). While there are some fibrils that consist of single protofilaments (e.g., Van Melckebeke et al., 2010; Tuttle et al., 2016; Radamaker et al., 2019; Schmidt et al., 2019; Swuec et al., 2019; Lu et al., 2020; Li et al., 2021; see Figures 4–6 for examples and Figure 9A for a schematic), instances of multiple protofilaments appear to be somewhat more common (e.g., Lührs et al., 2005; Paravastu et al., 2008; Fitzpatrick et al., 2017; Gremer et al., 2017; Iadanza et al., 2018b; Li et al., 2018a; Kollmer et al., 2019; Röder et al., 2020; Schweighauser et al., 2020; Cao et al., 2021; see Figures 5, 6 for examples and Figures 9B–E for schematics), and these fibrils usually have a well-defined symmetry and set of interactions between protofilaments. In this section, we discuss the various modes of packing, and the interactions and general structural principles responsible for supra-protofilament assembly.

### 5.1. Symmetry of Protofilament Association

Amyloids exhibit a wide variety of protofilament packing symmetries, and, alongside subunit structure, this is one of the main sources of polymorphism. Fibrils most often consist of a pair of protofilaments (Figure 9B), but associations of three (e.g., Paravastu et al., 2008; Hervas et al., 2020; Bansal et al., 2021; Figure 9C) or four (e.g., Jiménez et al., 1999, 2002; Lührs et al., 2005; Schmidt et al., 2009; Lattanzi et al., 2021; Yang et al., 2022; Figure 9D) protofilaments are also common, and larger numbers sometimes occur (e.g., Jiménez et al., 2002; Fitzpatrick et al., 2013; Close et al., 2018; Salinas et al., 2018; Figure 9E). In fibrils that consist of a small number of protofilaments, the protofilaments are typically related to one another by simple rotation about the fibril axis, forming cyclically symmetric fibrils that usually have a twisted ribbon or cylindrical morphology (Figures 9B–D). In more complex cases, protofilaments may be further organized into subgroups that occupy an intermediate level of the structural hierarchy between protofilaments and fibrils. For example, some twisted ribbon fibrils consist of four protofilaments that are organized as a twofold-symmetric association of protofilament pairs (e.g., Schmidt et al., 2015; Lattanzi et al., 2021; Yang et al., 2022; Figure 9D, see lower top-down schematic). However, not all twisted ribbon fibrils have rotational symmetry, and cases with asymmetric orientations or differing protofilament structures have been observed (e.g., Jiménez et al., 2002; Dearborn et al., 2016; Fitzpatrick et al., 2017; Schweighauser et al., 2020; Cao et al., 2021; see Figures 5G,J,N). Alternatively, protofilaments or groups of protofilaments may associate in a row, forming tape-like structures (e.g., Lührs et al., 2005; Fitzpatrick et al., 2013; Zhang et al., 2013; Adamcik et al., 2016; Seuring et al., 2017; Close et al., 2018; Figure 9E) that either twist to form helices (Zhang et al., 2013; Seuring et al., 2017), or flatten out to form sheet-like structures similar to 2D crystals (Adamcik et al., 2016; Reynolds et al., 2017). Supra-protofilament organization is a source of considerable polymorphism, with different fibril polymorphs differing not only in the number of protofilaments, but also their arrangement and mode of interaction. For example, there are at least four α-syn polymorphs that have a similar protofilament structure but a completely different set of packing interactions (Li et al., 2018a; Boyer et al., 2019), and a similar phenomenon has been reported for Aβ(1–40) (Paravastu et al., 2008; Meinhardt et al., 2009) and Tau (Fitzpatrick et al., 2017).

Protofilaments are polar structures, with the backbone hydrogen bonding groups oriented in a particular direction along the protofilament axis, and each end of the protofilament presenting a distinct interface for addition of new subunits. As a result, a pair of associated protofilaments can be oriented either parallel or antiparallel to one another. The parallel orientation is much more common; while antiparallel and mixed fibrils have been predicted in coarse-grained simulations (Pellarin et al., 2010), they do not appear to be well-attested in experimental structures. The bias toward parallel orientation may be partly driven by the self-assembly mechanism; as previously mentioned, the polar nature of protofilaments often results in unequal elongation rates at their two ends, with a bias toward elongation in a particular direction (Ban et al., 2004; Young et al., 2017). If two nascent protofilaments laterally associate in a parallel orientation, they will exhibit biased elongation in the same direction, allowing cooperative extension of the structure as a whole. If, stochastically, one protofilament should end up shorter than the other, the growth enhancement due to templating by the longer protofilament will eventually allow it to catch up, limiting the lifespan of any overhanging ends, and maintaining coordinated growth of the two protofilaments. On the other hand, if two protofilaments associate in an antiparallel orientation, they will exhibit biased elongation in opposite directions, causing one protofilament to lead at each end. As with the parallel orientation, the leading protofilament will probably template assembly of the trailing protofilament to some extent; however, in this case there is no guarantee that the resulting growth enhancement will be enough to maintain coordinated elongation, given the potential for a large disparity between the intrinsic growth rates of the two protofilaments. Therefore, coordinated growth of protofilaments may be harder to achieve in an antiparallel orientation, limiting the expansion of fibril segments that have that orientation. In protofilaments with a low growth polarity, the pressure for parallel orientation is not likely to exist; furthermore, successful association of a pair of protofilaments in this manner will result in an apolar fibril structure, with both ends of the fibril presenting the same pair of interfaces for elongation.

**Figure 10:**
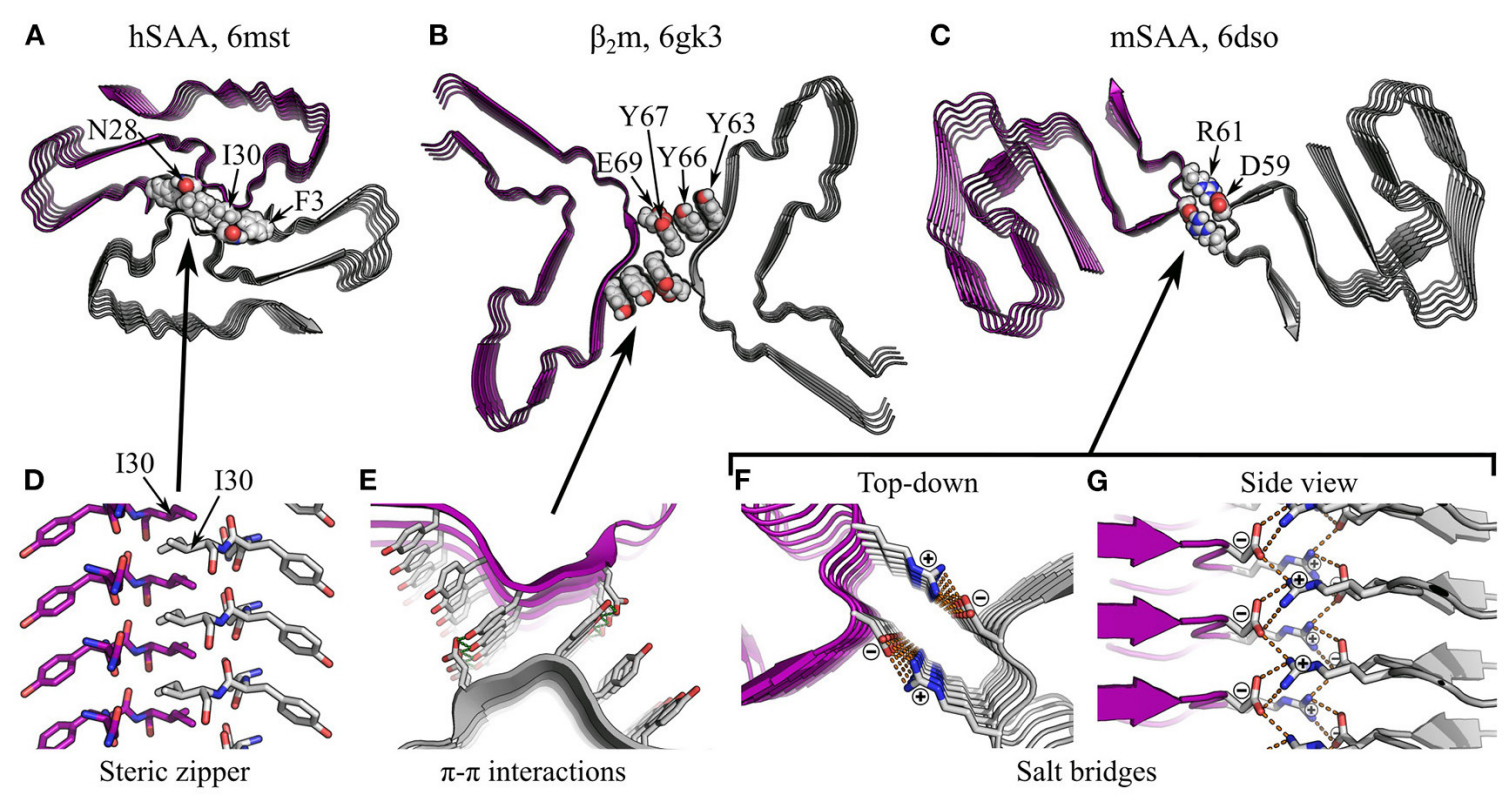
Specific interactions in supra-protofilament assembly (part 1). **(A–C)** show various fibril structures with specific inter-protofilament interactions highlighted: **(A)**, human serum amyloid A (hSAA; Liberta et al., 2019), with an inter-protofilament steric zipper; **(B)**, β_2_m (Iadanza et al., 2018b), with π-stacking by tyrosines and sidechain-sidechain hydrogen bonding between Y67 and E69; **(C)**, murine serum amyloid A (mSAA; Liberta et al., 2019), with salt bridges between D59 and R61. The name of the polypeptide is given above each structure, alongside the PDB ID. Structures are shown as ribbon diagrams, with protofilaments colored gray or purple for discrimination. Sidechains of interest are highlighted as spheres, with the color scheme: gray, carbon/hydrogen; red, oxygen; blue, nitrogen. For **(B)**, missing hydrogens have been modeled in. The same scale is used throughout **(A–C)**, and is shared with that used in Figures 11A,B. **(D–G)** show close-up views of the highlighted interactions. In **(E–G)**, structures are shown as ribbon diagrams with sidechains as sticks, using the same color scheme as before; **(D)** is a side view of the steric zipper in hSAA fibrils, shown entirely as sticks and with the carbons on one protofilament colored purple. For clarity and consistency between structures, hydrogens are not represented with sticks and are thus implicit. Hydrogen bonds are represented by green dashed lines, and salt bridges in mSAA are represented by orange dashed lines. Note that the zig-zag alternation of sidechains across the interfaces in **(D,G)** is due to pseudo-2_1_ symmetry.

**Figure 11:**
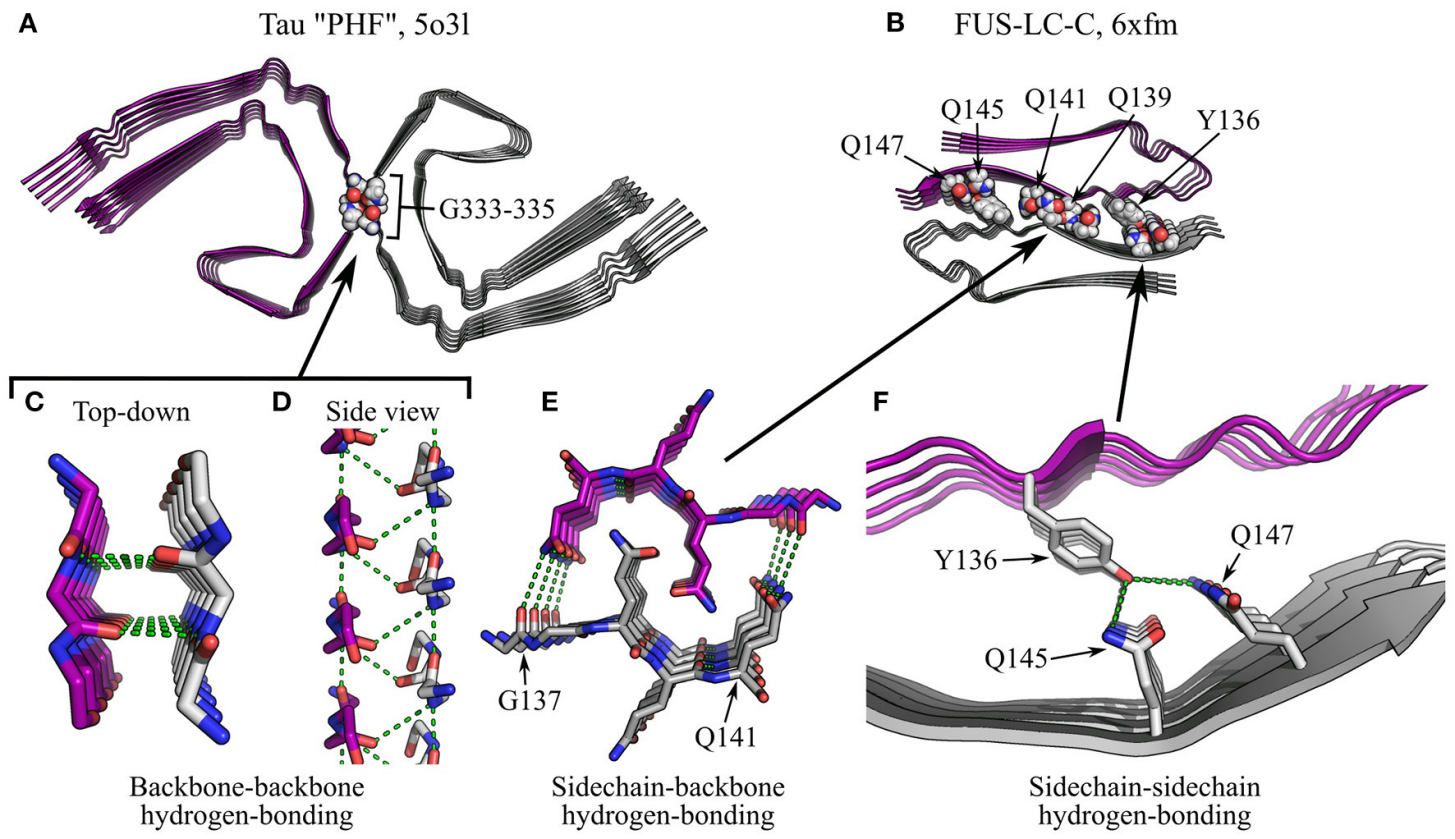
Specific interactions in supra-protofilament assembly (part 2). **(A,B)** show fibril structures with specific inter-protofilament interactions highlighted: **(A)**, Tau “PHF” polymorph (Fitzpatrick et al., 2017), with backbone-backbone hydrogen bonding by triglycine repeats; **(B)**, FUS-LC-C (Lee et al., 2020), with sidechain-backbone hydrogen bonding by interdigitated glutamines, and sidechain-sidechain hydrogen bonding by Y136, Q145, and Q147. The name of the polypeptide is given above each structure, alongside the polymorph in quotes where relevant, and the PDB ID. Structures are shown as ribbon diagrams, with protofilaments colored gray or purple for discrimination. Sidechains of interest are highlighted as spheres, with the color scheme: gray, carbon/hydrogen; red, oxygen; blue, nitrogen. For **(B)**, missing hydrogens have been modeled in. The same scale is used throughout **(A,B)**, and is shared with that used in Figures 10A–C. **(C–F)** show close-up views of the highlighted interactions. In **(F)**, the structure is shown as a ribbon diagram with the sidechains as sticks, using the same color scheme as before; in **(C–E)**, both the backbone and sidechains are shown as sticks, with carbons on one protofilament colored purple. For clarity and consistency between structures, hydrogens are not represented with sticks and are thus implicit. Hydrogen bonds are represented by green dashed lines. Note that the zig-zag alternation of polypeptide backbones across the interface in **(D)** is due to pseudo-2_1_ symmetry.

The structure of a fibril is typically maintained by a well-defined set of interactions between its constituent protofilaments. Early models assumed an in-plane alignment between adjacent subunit stacks (Paravastu et al., 2008; Schütz et al., 2015; Wälti et al., 2016); however, with the advent of high-resolution cryo-EM density maps that give more precise information about the relative orientation of the protofilaments, it has become clear that many fibrils that consist of a pair of protofilaments have a pseudo-2_1_ screw symmetry (e.g., Fitzpatrick et al., 2017; Gremer et al., 2017; Guerrero-Ferreira et al., 2018, 2019; Li et al., 2018a,b; Kollmer et al., 2019; Liberta et al., 2019; Röder et al., 2019; Glynn et al., 2020; Zhao et al., 2020; Bansal et al., 2021; Cao et al., 2021; Yang et al., 2022; see Figure 9B for a schematic, and Figures 10, 11 for examples of the protofilament packing interfaces of such fibrils). In this very common arrangement, one of the subunit stacks is ~2.4 Å further along the fibril axis than the other, half of the ~4.8 Å separation between β-strands in a single stack (Figures 9–11). Thus, the complete fibril can be analyzed as a 2_1_ screw, in which each monomer is separated from the “last” by a translation of ~2.4 Å along the central fibril axis and a rotation of ~180^o^ about that axis. Although in-plane alignments also occur for rotationally symmetric twofold fibrils, giving an overall C_2_ symmetry (e.g., Fitzpatrick et al., 2017; Iadanza et al., 2018b; Cao et al., 2019, 2021; Bansal et al., 2021; Yang et al., 2022), pseudo-2_1_ symmetry appears to be somewhat more common, perhaps due to the enthalpic advantages of improved packing at the interface between protofilaments (Figures 10, 11). Nonetheless, the overall symmetry is still influenced by system-specific constraints, and several instances will be presented in the following paragraphs where the geometry of key interactions at the interface appears to favor a particular arrangement.

### 5.2. Hydrophobic Interactions Between Protofilaments

For both rotationally symmetric and screw symmetric fibrils, the high degree of alignment between protofilaments allows a specific set of molecular interactions to occur at their interface. These interactions are typically more similar to those responsible for subunit folding than subunit stacking, although there are some interesting exceptions. Many protofilament interfaces are desolvated; unsurprisingly, the hydrophobic effect and van der Waals interactions play an important role in this context. As with subunit folding, steric zippers are also a very common feature of such interfaces (e.g., Madine et al., 2008; Schmidt et al., 2015; Krotee et al., 2017; Guerrero-Ferreira et al., 2018; Li et al., 2018a,b; Liberta et al., 2019; Glynn et al., 2020; Zhao et al., 2020; see Figures 6N,O, 10A,D for examples), and their formation between protofilaments seems to be driven by broadly similar principles to their formation within protofilaments (see Section 4.1). In addition, more unusual interactions may occur. For example, a cryo-EM structure of fibrils formed by β_2_-microglobulin (β_2_m) has a protofilament packing interface stabilized by π-stacking (Iadanza et al., 2018b). In this structure, which consists of a pair of protofilaments with an overall C_2_ symmetry, the interface between each pair of laterally apposed subunits contains a stack of six tyrosines, three from each protofilament, oriented orthogonal to the protofilament axis (Figures 10B,E). While the distance between tyrosines across the cleft is sub-optimal for π-stacking, and an inter-protofilament hydrogen bond between Y67 and E69 is also present, the structure raises the possibility that π-π interactions, and perhaps also π-amide interactions, might contribute to supra-protofilament assembly (Iadanza et al., 2018b). This unusual interface may explain the C_2_ symmetry of the β_2_m structure, as the lack of a requirement for interdigitation and the dependence of the dominant interactions on alignment of subunits in the same plane would favor a C_2_ symmetry over a pseudo-2_1_ screw. More generally, the comparative weakness of this interface also demonstrates the principle that inter-protofilament interfaces need not be particularly strong to hold protofilaments together, as they occur in large numbers along the length of the fibril and thus have a high avidity, and they are also stabilized by steric constraints resulting from helical twisting of the protofilaments around one another (Iadanza et al., 2018b).

### 5.3. Salt Bridges and Hydrogen Bonding Between Protofilaments

Arrays of polar interactions can also stabilize the interfaces between protofilaments. Many interfaces, dry or wet, are stabilized by salt bridges between ladders of charged sidechains or termini (e.g., Schütz et al., 2015; Gremer et al., 2017; Close et al., 2018; Guerrero-Ferreira et al., 2018; Li et al., 2018a,b; Kollmer et al., 2019; Liberta et al., 2019; Lattanzi et al., 2021; Yang et al., 2022). In fibrils with a pseudo-2_1_ screw symmetry, the alignment of the subunits of one protofilament with the stacking interfaces between the subunits of the other encourages charged groups in these ionic ladders to form salt bridges with oppositely charged residues above and below them on the opposing protofilament, creating a zig-zag arrangement similar to a dipolar chain (Liberta et al., 2019; Figures 10C,F,G). Similarly, many inter-protofilament interfaces are stabilized by hydrogen bonding. For example, in the paired helical filaments (PHFs) of Tau, a pseudo-2_1_ screw interface is stabilized by backbone-backbone hydrogen bonding between triglycine repeats (Fitzpatrick et al., 2017; Figures 11A,C,D). Here, the residues G333, G334, and G335 adopt a polyglycine-II spiral conformation, causing backbone hydrogen bonding groups to point into the cleft (Figure 11C). When combined with the pseudo-2_1_ symmetry of the PHFs, this allows the formation of an alternating, zig-zag network of hydrogen bonds that holds the protofilaments together (Figure 11D). In addition, many fibrils are stabilized by inter-protofilament sidechain-backbone hydrogen bonding, such as a supplementary hydrogen bond from the sidechain amide of Q336 to the backbone carbonyl of K331 in PHFs (Fitzpatrick et al., 2017), a bond from the sidechain amide of Q141 to the backbone carbonyl of G137 in FUS-LC-C fibrils (Lee et al., 2020; Figures 11B,E), and a bond from the hydroxyl of Y169 to the backbone carbonyl of N171 in proto-PrP^Sc^ microcrystals (Gallagher-Jones et al., 2018). Lastly, sidechain-sidechain hydrogen bonds are also observed, and include the Y67-E69 hydrogen bond in β_2_m fibrils (Iadanza et al., 2018b; Figures 10B,E), hydrogen bonding between Y136, Q145, and Q147 in FUS-LC-C fibrils (Lee et al., 2020; Figures 11B,F), and various bonds between asparagine, glutamine, and serine in designer peptides (Wang et al., 2018; Peccati et al., 2020). As with similar interactions within protofilaments (Section 4.2), the capacity of polar moieties to simultaneously form multiple salt bridges or hydrogen bonds allows the assembly of repetitive arrays of mutually supporting interactions (e.g., Figures 10G, 11D), strengthening the structure of individual protofilaments and the fibril as a whole.

### 5.4. Hydrated Channels

An interesting feature of some amyloid fibrils is the presence of hydrated channels running along their interior. Although water-filled cavities do sometimes occur within individual protofilaments, such as the case of α-syn (Guerrero-Ferreira et al., 2018, 2019; Li et al., 2018a,b; Boyer et al., 2019), these are typically narrow and the water molecules within tend to be relatively ordered. Between protofilaments, however, much larger channels can form, and these may be lined by hydrophilic or hydrophobic sidechains. For example, the “3Q” polymorph of Aβ(1–40) (Paravastu et al., 2008), which is a rotationally symmetric fibril with three protofilaments, has a dry interface between the protofilaments around the outside of the fibril, but a hollow core at the center with a hydrophobic lining (Miller et al., 2011; McDonald et al., 2012). A central channel has been observed in amyloid fibrils formed by a wide variety of other polypeptides, including transthyretin, SH3 amyloid, amyloid A, and Aβ(1-42) (Serpell et al., 1995, 2000; Jiménez et al., 1999; Zhang et al., 2009). These channels typically have an elliptical cross-section, so that they are wider in one axis than in the other, are structurally unrelated to those found in pore-forming amyloid oligomers, and have different dimensions, as oligomer pores have a typical length of 4–5 nm and inner diameter of 1–2 nm (Quist et al., 2005; Jang et al., 2010), while channels in amyloid fibrils are much longer, and can have a diameter of up to ~4 nm in the narrowest axis (Zhang et al., 2009), which is large enough to accommodate a small globular protein. Although fibril channels may be able to sequester or transport a wide variety of molecules, to our knowledge such activity has yet to be demonstrated, and their potential role in function or pathology remains unclear.

## 6. Mesoscale Structural and Mechanical Properties

The interactions that maintain subunit stacking, folding, and supra-protofilament assembly are highly sensitive to differences in conformation and orientation between adjacent monomers. As a result, amyloids exhibit an unusual degree of long-range order, and the conformation and orientation of their constituent monomers remain strongly correlated over large length scales (typically several microns). This gives amyloids structural and mechanical properties unlike those of most other protein aggregates, in which the orientation and often also conformation of assembled monomers decorrelate over a matter of nanometers, resulting in an amorphous structure. In particular, amyloids are notable for their mesoscale chirality, rigidity, and high tensile strength. Viewed by EM or AFM, amyloids are often visibly chiral, with a helical or twisted ribbon topology, and a strong correlation in their pitch or twist rate along their length (Knowles et al., 2006). While some are flexible, meaning that the persistence length *l*_*p*_ over which the direction of the fibril axis decorrelates is much less than their typical length (*l*_*p*_≪*l*), many maintain the same direction across their length and are thus relatively rigid (*l*_*p*_≫*l*) (Knowles et al., 2007; Yagi et al., 2007); in addition, they often have high tensile strength (Smith et al., 2006; Sweers et al., 2012). The rigidity and tensile strength of amyloids are testament to the stable, extensive network of interactions that maintains their structure, and the low frequency of structural defects. The nanoscale structure of amyloids is inextricably related to their mesoscale properties, meaning that small changes in the former can dramatically affect the latter; in this section, we outline the factors that contribute to these properties, and discuss the mechanical characteristics of amyloids in the context of other materials and biomacromolecules.

### 6.1. Mesoscale Chirality of Amyloid Fibrils

Amyloids tend to have left-handed helical or twisted ribbbon topologies, although right-handed and achiral topologies are also observed (e.g., Reynolds et al., 2017; Kollmer et al., 2019; Liberta et al., 2019; Aubrey et al., 2020). The molecular-level chirality of the constituent polypeptide chains is only able to propagate to the mesoscale level because of the stable, uniform, and highly repetitive interactions between stacked subunits, and at the interface between protofilaments. In parallel in-register structures, there is a close, two-way relationship between the chirality of the protofilament and that of its constituent β-strands, so that the preferences of the polypeptide chain can affect the mesoscale morphology, and *vice versa*. Generally speaking, β-sheets formed from β-strands with a right-handed twist along their length will tend to twist in a left-handed manner between strands, and this in turn results in a fibril with a left-handed chirality; conversely, β-strands that have a left-handed twist will result in a protofilament with a right-handed twist, as confirmed by existing amyloid structures (Kollmer et al., 2019; Liberta et al., 2019). Due to unfavorable interactions between the backbone carbonyl and the sidechain, non-amyloid proteins strongly prefer right-handed β-strands (Chothia, 1973; Lovell et al., 2003), and this probably also explains the tendency of amyloids to form left-handed protofilaments. Nonetheless, right-handed protofilaments are also observed, and the same polypeptide may form fibril polymorphs with different chirality depending on the formation conditions, or even concurrently during polymorphic self-assembly (Kollmer et al., 2019; Aubrey et al., 2020). Thus, while the twisting of protofilaments is coupled to that of their constituent β-strands, neither is solely determined by the primary sequence, and extrinsic factors can affect both. In particular, while backbone-backbone interactions tend to limit twisting, sidechain interactions tend to encourage it (Periole et al., 2018). Repulsion between stacked electrostatics appears to be particularly important in inducing torsion, meaning that factors such as pH and ionic strength can alter fibril morphology; for example, fibrils of β-lactoglobulin and β-endorphin have a twisted appearance when grown at low ionic strength, and a flat, ribbon-like appearance at high ionic strength (Adamcik and Mezzenga, 2011; Assenza et al., 2014; Seuring et al., 2017). Interactions between protofilaments are also important, presumably because of the need to modify chirality to optimize inter-protofilament interactions, and protofilament association is usually accompanied by a change in the rate of twist (Meinhardt et al., 2009; Schmidt et al., 2009; Adamcik et al., 2010; Li et al., 2018a; Boyer et al., 2019; Röder et al., 2020), and occasionally even handedness (Usov et al., 2013). Lastly, there is an inverse correlation between the fibril width and twisting rate due to the greater shear stress experienced by wider fibrils, so that fibrils whose protofilaments are thicker, more numerous, or distributed further from the central axis will tend to have a lower rate of twist (Meinhardt et al., 2009). For particularly wide fibrils, a twisted ribbon topology becomes unsustainable, and protofilaments instead form helically coiled, tape-like structures, as the shear stress in these structures is less closely related to the number of protofilaments (Reynolds et al., 2017).

### 6.2. Rigidity and Tensile Strength

The same structural properties affect fibril rigidity. When a fibril is bent in a particular direction, its bending stiffness is proportional to the planar second moment of area in that axis, *I*, which also strongly depends on the width of the fibril (Riley et al., 2006). Thus, thinner fibrils will bend more easily in response to thermal fluctuations, and will have a more curvilinear appearance when viewed by EM or AFM, whereas thicker fibrils will tend to have a more rod-like appearance. The persistence length of a fibril is given by the relation *l*_*p*_ = *EI*/*k*_*B*_*T*, where *E* is the Young's modulus, *I* is likely to be dominated by the lowest-energy bending mode, *k*_*B*_ is Boltzmann's constant, and *T* is the temperature (Landau and Lifshitz, 1979). In general, the dependence of *I* on the fibril width is approximately *I*~*w*^4^, so that even small variations in width can strongly affect rigidity; coupled with significant variation in total length, from tens of nm to tens of μm, this means that the morphology of amyloid fibrils can vary from flexible (*l*_*p*_≪*l*) to rod-like (*l*_*p*_≫*l*) (Knowles et al., 2007; Yagi et al., 2007). These differences are mainly attributable to variations in the size, number, and packing of protofilaments, and comparisons of a variety of fibrils have revealed a relatively narrow range of Young's moduli, 2–14 GPa, implying underlying structural commonalities (Knowles et al., 2007). Lastly, amyloids typically have a tensile strength in the 0.1–1 GPa range; this is in the same range as steel, and testifies to the uniformity of the fibril structure, the low rate of defects, and the strong network of interactions that maintains it (Smith et al., 2006; Sweers et al., 2012). Overall, the mechanical properties of amyloids make them highly attractive for materials science applications. In addition, they are of direct relevance to physiology, as they control the rate of fragmentation, which promotes prion-like spreading and toxicity (Xue et al., 2009; Wang et al., 2011), and directly affect the efficacy of functional amyloids that perform a structural role, such as the Gram-negative bacterial amyloid curli, which is involved in cell adhesion and biofilm formation (Chapman et al., 2002).

## 7. Discussion

### 7.1. General, Amyloid-Specific Principles of Structural Organization

Amyloids are structurally diverse, but have shared characteristics that differ markedly from those of globular proteins and point to amyloid-specific, unifying structural principles. As a consequence of cross-β structure, particular shared characteristics include: (i) the open-endedness and scale of self-assembly; (ii) the potential for self-replication and seeding; (iii) a hierarchical organization, where different symmetries and interaction motifs predominate in maintaining stacking parallel to the fibril axis, compaction of subunits orthogonal to the fibril axis, and lateral association of protofilaments; (iv) the importance of ladder-like interaction motifs in subunit stacking; (v) the predominance of distinctive zipper-like motifs in subunit folding and supra-protofilament assembly; (vi) the recurrence of certain protofilament packing symmetries; and (vii) unusual mesoscale properties such as long-range order, chirality, and tensile strength.

Ultimately, the unusual characteristics of amyloids can be traced back to the peculiarities of cross-β structure itself. Unlike globular proteins, in which the hydrophobic effect mediates a comparatively isotropic collapse of the polypeptide chain, amyloids exhibit a coordinated alignment of backbone hydrogen bonding groups along a shared axis, allowing open-ended self-assembly and conformational replication. The alignment of hydrogen bonding groups and the unidirectional nature of hydrogen bonds are responsible for the filamentous structure of protofilaments (Fitzpatrick et al., 2011), and in turn this creates distinct assembly modes along and orthogonal to the fibril axis, resulting in a hierarchical organization and a differentiation between molecular interactions at different levels of this hierarchy. On the one hand, subunit stacking favors open-ended, ladder-like interactions such as π-stacking and amide ladder hydrogen bonding (Perutz et al., 1994; Gazit, 2002; Nelson et al., 2005), which complement the geometry and extensibility of the cross-β structure, and explain the strong preference of amyloids for parallel in-register or pseudo-in-register alignment. On the other hand, subunit folding and supra-protofilament assembly are dominated by a different set of interaction motifs. Broadly speaking, the segregation and packing of sidechains within amyloid fibrils can be explained in terms of the same principles that govern folding of globular proteins, applied to the pseudo-two-dimensional environment of stacked subunits. In this context, zipper-like interactions are inevitable, due to the formation and subsequent pairing of repeating ladders of functional groups along the length of cross-β structures. While steric zippers (Nelson et al., 2005) are particularly common, and appear to be the amyloids' equivalent of desolvated core formation in globular proteins, they are part of a broader picture that also includes salt bridge formation between ionic ladders (e.g., Schütz et al., 2015; Gremer et al., 2017; Close et al., 2018; Guerrero-Ferreira et al., 2018; Li et al., 2018a,b; Kollmer et al., 2019; Liberta et al., 2019), lateral hydrogen bonding by amide ladders (Gallagher-Jones et al., 2018; Wang et al., 2018; Hervas et al., 2020; Lee et al., 2020; Peccati et al., 2020), backbone-backbone hydrogen bonding (Fitzpatrick et al., 2017), and the π-stacked interface of β_2_m fibrils (Iadanza et al., 2018b). These motifs are all specific realizations of the same general principle, that the formation of cross-β structure favors stacking of alike or complementary residues along a single axis, and the usual rules of protein folding induce segregation, packing, and zipper formation of those residues orthogonal to that axis. An additional consequence of the hierarchical organization and regularity of amyloids is that interactions in different axes tend to link up to form repeating multidimensional networks that further stabilize the structure, such as the formation of tetragonal hydrogen bonding networks by amide ladders (Gallagher-Jones et al., 2018; Hervas et al., 2020; Figures 7G,I), the zig-zag alternation of polar sidechains in FUS-LC-C and mSAA fibrils (Liberta et al., 2019; Lee et al., 2020; Figures 7H, 10G), and the triangular pattern of backbone-backbone hydrogen bonding at the interface of Tau PHFs (Fitzpatrick et al., 2017; Figure 11D). The regularity and cooperativity of interactions in amyloid fibrils allow molecular order to be maintained over large length scales and the chirality and strength of the cross-β structure to be reflected at the mesoscale level, resulting in the unusual mechanical and functional properties of amyloid fibrils.

### 7.2. Shared Structural Features Imply Common Structure-Activity Relationships

The universality and shared structural principles of amyloid self-assembly imply a similar degree of commonality in their structure-activity relationships. In agreement with this, amyloids formed by diverse polypeptides exhibit striking similarities in their mechanisms of self-assembly and pathogenesis. These include: (i) a capacity for seeding and prion-like spreading (Jarrett and Lansbury, 1993); (ii) a tendency for pathogenic amyloids to have a highly stable core, whereas many functional amyloids exhibit adaptations to reduce core stability (Sawaya et al., 2021); (iii) a nucleated polymerization mechanism of formation (Jarrett and Lansbury, 1992; Come et al., 1993); (iv) a tendency to nucleate in oligomeric or droplet-like intermediates that are often rich in β-structure (e.g., Serio et al., 2000; Bitan et al., 2001; Chimon et al., 2007; Thakur et al., 2009; Lee et al., 2011; Molliex et al., 2015; Shammas et al., 2015; Iljina et al., 2016; Ambadipudi et al., 2017; Yang et al., 2018; Ray et al., 2020; Ashami et al., 2021); (v) the toxicity of diverse amyloid-related oligomers, and some amyloid fibrils (e.g., Lambert et al., 1998; Tucker et al., 1998; Rochet et al., 2000; Mukai et al., 2005; Quist et al., 2005; Xue et al., 2009; Milanesi et al., 2012; Kollmer et al., 2016; Schützmann et al., 2021); (vi) the capacity of both mature amyloids and oligomers to disrupt lipid membranes (e.g., Rhee et al., 1998; Quist et al., 2005; Kayed et al., 2009; Xue et al., 2009; Jang et al., 2010; Milanesi et al., 2012; Kollmer et al., 2016; Flagmeier et al., 2020); (vii) and the ability of amyloids to induce further aggregation and toxicity by secondary nucleation (Ruschak and Miranker, 2007; Andersen et al., 2009; Mizuno et al., 2011; Cohen et al., 2013; Gaspar et al., 2017; Frankel et al., 2019). Just as the structural similarities between amyloid fibrils point to shared principles of self-assembly, their behavioral similarities point to shared structure-activity principles.

The capacity for prion-like spreading has long been recognized as a consequence of the ability of fibril ends to template structural conversion of non-amyloid precursors to an amyloid state (Jarrett and Lansbury, 1993). Without tight regulation, open-ended conformational self-replication poses obvious risks to organisms, due to the possibilities of mechanical damage due to widespread aggregation, altered activity or loss of function of the amyloid state, and generic, sequence-independent toxic effects such as membrane damage (Xue et al., 2009; Milanesi et al., 2012; Kollmer et al., 2016). As a result, the association between core stability and pathogenicity has been attributed to the hazards of irreversible deposition (Sawaya et al., 2021). Conversely, many functional amyloids have reduced core stability, which helps to avoid toxic accumulation and also allows rapid, function-specific disassembly. For example, storage amyloids such as FUS or β-endorphin have to be able to disassemble readily when the soluble form of the protein is required. A number of modifications can reduce the stability of the fibril core, including enrichment of the core-forming regions of the protein with hydrophilic residues that are easily solvated (Lu et al., 2020; Sawaya et al., 2021), primary sequence changes that reduce the contact area in intra-protofilament and inter-protofilament packing interfaces (Hughes et al., 2018; Sawaya et al., 2021), and the inclusion of post-translationally modifiable, cofactor-binding, or pH-responsive sequence motifs, which allow adaptive changes in the core stability in response to environmental stimuli (McGlinchey and Lee, 2017; Murray et al., 2017; Seuring et al., 2020). Thus, although studies of the structure-activity relationships of functional amyloids are still at an earlier stage than those of pathogenic amyloids, it appears there may be general sequence determinants of the balance between functionality and pathogenicity (Sawaya et al., 2021).

Nucleated polymerization implies that there is a free energy cost associated with the early stages of amyloid formation, which is not present during the growth of larger, more structurally mature amyloids. In turn, this may be largely due to the the self-stabilizing nature of subunit stacking in longer protofilaments (Tsemekhman et al., 2007), the entropic barrier for desolvated core formation (Nelson et al., 2005; Reddy et al., 2010), and a high degree of cooperativity resulting from the three-dimensional structure of amyloids (Zhang and Schmit, 2016) and the complex, extensive networks of interactions highlighted in this review. While the tendency to nucleate via partly ordered intermediates is shared with diverse crystallization processes (e.g., ten Wolde and Frenkel, 1997; Gavezzotti, 1999; Shore et al., 2000; Nicolis and Nicolis, 2003), the abundance of intermediates that lack either cross-β structure or a mature subunit fold suggests that the hierarchical organization of amyloids may lead to distinct free energy barriers associated with successive stages of self-assembly (Serio et al., 2000; Yong et al., 2002; Krishnan and Lindquist, 2005; Chimon et al., 2007; Thakur et al., 2009; Ahmed et al., 2010; Sandberg et al., 2010; Urbanc et al., 2010; Dupuis et al., 2011; Buchanan et al., 2013; Zheng et al., 2016; Brown et al., 2018; Ray et al., 2020). However, the precise mechanisms of amyloid nucleation are still a matter of debate, and it is likely that there is more than one sequence of structural transitions that can lead to the amyloid state.

The oligomers that are formed as intermediates or by-products of amyloid nucleation can also have generic mechanisms of toxicity, and, consequently, functional amyloids are suggested to experience a pressure to minimize oligomerization (Dear et al., 2020). One of the best candidates for generic toxicity by both mature amyloids and amyloid-related oligomers is disruption of lipid membranes. Mature amyloids typically induce membrane distortions via exposed hydrophobics at their ends (Xue et al., 2009; Milanesi et al., 2012; Kollmer et al., 2016), a natural consequence of the pseudo-planar structure of their subunits; however, they can also interact with lipids along their length (Kollmer et al., 2019), or even co-assemble with lipids (Galvagnion et al., 2019). Globular oligomers and certain metastable amyloid fibrils appear to exhibit a generic capacity to transition to an amyloid-like β-barrel state (Jang et al., 2008, 2010; Bellesia and Shea, 2009; Kayed et al., 2009; Tomic et al., 2009; Laganowsky et al., 2012; Liu et al., 2012), and these can act as membrane pores (Quist et al., 2005; Jang et al., 2008, 2010; Mustata et al., 2009); in addition, other forms of oligomer-dependent membrane disruption have been documented (Green et al., 2004; Kayed et al., 2004). Apart from membrane interactions, it is worth noting that amyloid fibrils provide a generic mechanism by which functional proteins can undergo a pathological loss of function. In addition, any activity retained after assembly into the amyloid state could be strongly affected by the high avidity of amyloid fibrils, potentially resulting in dysregulation and toxicity.

Lastly, secondary nucleation remains a topic of active research, but general mechanistic principles are beginning to emerge. Several studies have implicated attractive interactions between monomers or oligomers and the fibril surface in increasing the local concentration of protein (Barz and Strodel, 2016; Šarić et al., 2016; Bunce et al., 2019), and the distinct environment of the fibril surface may favor structure formation compared to the bulk solvent (Barz and Strodel, 2016; Bunce et al., 2019). Stacks of exposed hydrophobic residues may play a particularly important role in binding and folding (Barz and Strodel, 2016); in addition, there may also be a specific templating effect resulting from complementarity between the existing and nascent fibril structures. In line with this, we note that many amyloids, such as Aβ (Paravastu et al., 2008; Xiao et al., 2015; Colvin et al., 2016; Wälti et al., 2016; Gremer et al., 2017; Yang et al., 2022), α-syn (Li et al., 2018a; Boyer et al., 2019), and Tau (Fitzpatrick et al., 2017), have several distinct choices of protofilament binding interface, which may allow assembly of fibril nuclei along the unoccupied interfaces of mature fibrils. Thus, amyloids exhibit generic mechanisms of self-assembly and activity that are intimately related to their shared structural characteristics, such as their open-endedness, hierarchical organization, and composition from stacked, pseudo-planar subunits.

### 7.3. Concluding Remarks

The existence of overarching principles that govern amyloid structure, self-assembly, and activity helps to explain the similarities between different amyloid diseases, indicates that insights acquired by studying one particular system are likely to be translatable to a wide variety of other amyloids, and suggests that there may be broad strategies to harness the functional properties of amyloids, and treat diseases caused by amyloid formation. While the recent explosion of high-resolution structural models has shown that the conformations of amyloids differ between *ex vivo* and *in vitro* sources (Kollmer et al., 2019; Schmidt et al., 2019; Zhang et al., 2019; Schweighauser et al., 2020; Bansal et al., 2021; Yang et al., 2022), this intricate new picture has also revealed a wealth of shared characteristics, many of which were previously unresolvable. Thus, while *ex vivo* structures are likely to be essential for targeted therapeutic development, studies of peptide models or *in vitro* amyloids have made crucial contributions to our understanding of amyloid structure, and continue to do so. For example, early studies of synthetic and recombinant amyloids demonstrated the prevalence of parallel in-register alignment (Blake and Serpell, 1996; Benzinger et al., 1998; Antzutkin et al., 2000), provided the first atomic-resolution models of subunit conformation (Petkova et al., 2002; Lührs et al., 2005; Paravastu et al., 2008; Wasmer et al., 2008), demonstrated the existence of crucial interaction motifs such as π-stacked aromatics (Gazit, 2002; Makin et al., 2005; Nelson et al., 2005), amide ladders (Chan et al., 2005; Nelson et al., 2005; Wasmer et al., 2008), and steric zippers (Nelson et al., 2005; Sawaya et al., 2007), laid the foundations of our understanding of supra-protofilament self-assembly (Blake and Serpell, 1996; Jiménez et al., 2002; Petkova et al., 2002; Lührs et al., 2005; Paravastu et al., 2008; Meinhardt et al., 2009; Schmidt et al., 2009; Fitzpatrick et al., 2013), and revealed the extent and malleability of amyloid polymorphism (Jiménez et al., 2002; Heise et al., 2005; Paravastu et al., 2008; Meinhardt et al., 2009; Qiang et al., 2013). Work on such systems continues to uncover novel interaction motifs, subunit folds, packing modes, and other structural features that anticipate or allow a better understanding of physiological amyloids (e.g., Tuttle et al., 2016; Gremer et al., 2017; Gallagher-Jones et al., 2018; Guerrero-Ferreira et al., 2018; Iadanza et al., 2018b; Li et al., 2018a,b, 2021; Lee et al., 2020; Röder et al., 2020), and ongoing *in vitro* investigations of polymorphism are likely to play a crucial role in our understanding of the determinants of physiological amyloid structure in the coming decade. Furthermore, the existence of small molecules and proteins with generic amyloid-binding or amyloid-modifying capabilities, including dyes such as Congo red and ThT (Bennhold, 1922; LeVine, 1993), polyphenols such as epigallocatechin gallate (EGCG; Ehrnhoefer et al., 2006; Rambold et al., 2008; Roberts et al., 2009; Bieschke et al., 2010), various chaperones (Shorter and Lindquist, 2004; DeSantis et al., 2012; Gao et al., 2015; Scior et al., 2018), and the bacteriophage protein G3P (Krishnan et al., 2014), suggests that it may be possible to develop therapeutics that generically target amyloids, or target structural sub-classes that exhibit particular activities. Thus, studies of diverse amyloids allow derivation of general structure-activity principles that help to explain how and why amyloids form, shed light on the environmental and structural determinants that cause function or pathogenicity, and instill hope in efforts to develop broad-spectrum modifiers of pathological self-assembly that can be used to restore proteostasis in diverse diseases, including those complicated by sequence or structural polymorphism.

## Author Contributions

AT wrote the draft. AT and RS edited the draft. Both authors approved the submitted version.

## Funding

The authors gratefully acknowledge financial support from the University of Sheffield and the BBSRC (grant no. BB/P002927/1).

## Conflict of Interest

The authors declare that the research was conducted in the absence of any commercial or financial relationships that could be construed as a potential conflict of interest.

## Publisher's Note

All claims expressed in this article are solely those of the authors and do not necessarily represent those of their affiliated organizations, or those of the publisher, the editors and the reviewers. Any product that may be evaluated in this article, or claim that may be made by its manufacturer, is not guaranteed or endorsed by the publisher.
